# Aberrantly high activation of a FoxM1–STMN1 axis contributes to progression and tumorigenesis in FoxM1-driven cancers

**DOI:** 10.1038/s41392-020-00396-0

**Published:** 2021-02-01

**Authors:** Jun Liu, Jipeng Li, Ke Wang, Haiming Liu, Jianyong Sun, Xinhui Zhao, Yanping Yu, Yihuan Qiao, Ye Wu, Xiaofang Zhang, Rui Zhang, Angang Yang

**Affiliations:** 1 State Key Laboratory of Cancer Biology, Department of Biochemistry and Molecular Biology, Fourth Military Medical University, 710032 Xi’an, Shaanxi China; 2 State Key Laboratory of Cancer Biology, Institute of Digestive Diseases, Xijing Hospital, Fourth Military Medical University, 710032 Xi’an, Shaanxi China; 3 Department of Experimental Surgery, Xijing Hospital, Fourth Military Medical University, 710032 Xi’an, Shaanxi China; 4 School of Software Engineering, Beijing Jiaotong University, 100044 Beijing, China; 5 Department of Thoracic Surgery, Tangdu Hospital, Fourth Military Medical University, 710038 Xi’an, Shaanxi China; 6 Department of Thyroid and Breast Surgery, Xi’an No. 3 Hospital, The Affiliated Hospital of Northwest University, 710018 Xi’an, Shaanxi China; 7 The Second Ward of Gynecological Tumor, Shaanxi Provincial Cancer Hospital, 710061 Xi’an, Shaanxi China; 8 School of Clinical Medicine, Xi’an Medical University, 710021 Xi’an, Shaanxi China; 9 State Key Laboratory of Cancer Biology, Department of Immunology, Fourth Military Medical University, 710032 Xi’an, Shaanxi China

**Keywords:** Gastrointestinal cancer, Tumour biomarkers

## Abstract

Fork-head box protein M1 (FoxM1) is a transcriptional factor which plays critical roles in cancer development and progression. However, the general regulatory mechanism of FoxM1 is still limited. STMN1 is a microtubule-binding protein which can inhibit the assembly of microtubule dimer or promote depolymerization of microtubules. It was reported as a major responsive factor of paclitaxel resistance for clinical chemotherapy of tumor patients. But the function of abnormally high level of STMN1 and its regulation mechanism in cancer cells remain unclear. In this study, we used public database and tissue microarrays to analyze the expression pattern of FoxM1 and STMN1 and found a strong positive correlation between FoxM1 and STMN1 in multiple types of cancer. Lentivirus-mediated FoxM1/STMN1-knockdown cell lines were established to study the function of FoxM1/STMN1 by performing cell viability assay, plate clone formation assay, soft agar assay in vitro and xenograft mouse model in vivo. Our results showed that FoxM1 promotes cell proliferation by upregulating STMN1. Further ChIP assay showed that FoxM1 upregulates STMN1 in a transcriptional level. Prognostic analysis showed that a high level of FoxM1 and STMN1 is related to poor prognosis in solid tumors. Moreover, a high co-expression of FoxM1 and STMN1 has a more significant correlation with poor prognosis. Our findings suggest that a general FoxM1-STMN1 axis contributes to cell proliferation and tumorigenesis in hepatocellular carcinoma, gastric cancer and colorectal cancer. The combination of FoxM1 and STMN1 can be a more precise biomarker for prognostic prediction.

## Introduction

Cancer is one of the high morbidity and mortality diseases. Although the survival rate of cancer patients has rapidly decreased in the past decades with deep understanding of the mechanisms of cancer initiation and progression, it is still an unconquered disease definitely. Strikingly, with growing body of evidence, researchers have summarized the major hallmarks of cancer, including sustaining proliferative signaling, evading growth suppressors, resisting cell death, enabling replicative immortality, inducing angiogenesis, activating invasion and metastasis, genome instability, inflammation, metabolic reprogramming and evading immune destruction. Of these ten hallmarks, excess growth is one of the most important characteristics of transformed cells, determining the fate of tumor cells.^1^ Aberrantly high activation of oncogenic transcriptional activators is closely associated with unlimited proliferation of transformed cells.

Fork-head box protein M1 (FoxM1) belongs to the Fox superfamily characterized by a conserved winged helix DNA-binding domain regulating cell division by transcription of a bundle of cell cycle-associated genes. It has been found that FoxM1 is frequently dysregulated in multiple cancers, and is emerged as an important molecule implicated in the initiation and progression of cancer. Functionally, it plays a vital role in the regulation of cancer cell proliferation and DNA damage repairment. A series of studies showed that aberrantly activated FoxM1 is closely associated with poor survival of clinical patients with breast cancer, ovarian cancer, bladder cancer, hepatocellular carcinoma, colorectal cancer^2^ and so on. And more strikingly, accumulating evidence has identified that a large number of downstream genes of FoxM1, such as CCNB1, CDC20, Cdc25B, Aurora B kinase, p21^cip1^, p27^kip1^, survivin, centromere protein A/B/F (CENPA, CENPB, CENPF) and so on, function as the oncogenic molecules to promote tumorigenicity.^2–6^ With more deep understanding of oncogenic roles and molecular mechanisms of FoxM1 in cancers, besides FoxM1-driven cell proliferation, other malignant behaviors such as angiogenesis,^7^ chemo-resistance,^8^ metastasis^9^ and even genome instability^10^ have been identified to be transcriptionally regulated by FoxM1 in multiple cancers. Exploring more molecular events regulated by FoxM1 will help us to further develop the potential cancer treatment strategy.

The dysfunctional assembly of microtubules causes mitotic disaster in cell mitosis and subsequently leads to cell death. This phenomenon provides a strategy targeting microtubule assembly to induce decreased cancer cell viability and even to suppress tumor growth in vivo. Stathmin1 (STMN1), also named as Oncoprotein 18, is a microtubule-binding protein, which can bind with α/β-Tubulin heterodimers, resulting in assembly suppression of microtubules, or promoting dissociation of microtubules.^11^ Recent studies have demonstrated that the high level of STMN1 can be detected in a variety of malignant tumor cells and is functionally related to promoting tumorigenicity and cancer metastasis.^12,13^ It has been reported that in multiple cancer cells, the high level of STMN1 antagonizes paclitaxel-induced mitotic disaster by promoting microtubule dissociation and cell division.^14^ Accordingly, STMN1 could be a potential target for the development of cancer therapeutics. And more importantly, though the high level of STMN1 frequently indicates poor prognosis in cancer patients, the underlying mechanism for high level of STMN1 in cancers is still elusive.

In the present study, we found that co-expression of FoxM1 and STMN1 is a frequent molecular event in multiple cancers. Furthermore, the co-expression pattern of these two molecules is closely associated with poor prognosis of almost all solid cancer patients derived from TCGA Database. Our mechanistic study showed that FoxM1 transcriptionally activates STMN1 in cancer cells using the promoter reporter assay, ChIP-qPCR and bioinformatics analysis in ENCODE ChIP-seq database. And more importantly, we found that the FoxM1–STMN1 axis is a general regulatory mechanism in multiple cancers. In the functional study, we investigated the biological effect of the FoxM1–STMN1 axis in multiple types of cancer cells using cell counting assay, clone formation assay, soft agar assay and xenograft nude mice model, and we found that the FoxM1–STMN1 axis contributes to cancer cell proliferation and tumor growth in vitro and in vivo. Our discovery here would shed light on the avenue to explore the new therapeutic strategy to target oncogenic pathways in cancers.

## Results

### Co-expression of FoxM1 and STMN1 in cancers

**Fig. 1:**
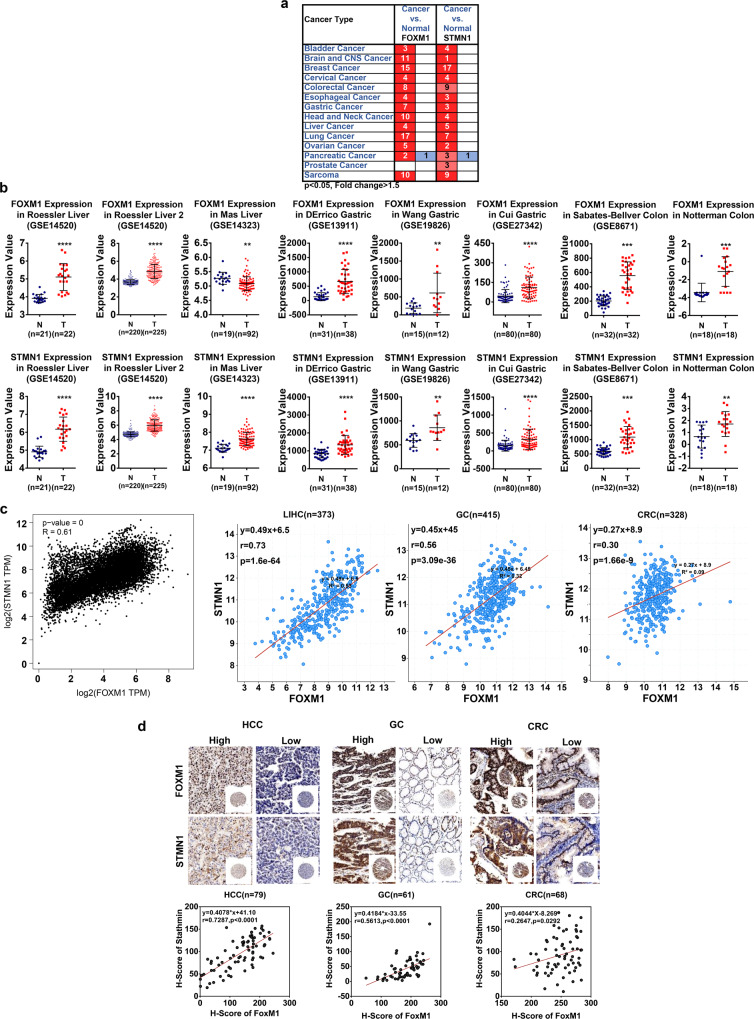
Coordinated expression of FoxM1 and STMN1 in cancers. **a** Analysis of the mRNA levels of FoxM1 and STMN1 (cancer vs. normal) in multiple solid cancers from Oncomine Database. **b** The expression of FoxM1 and STMN1 in hepatocellular carcinoma cohorts (GSE14520, GSE14323), gastric cancer cohorts (GSE13911, GSE19826, GSE27342) and colon cancer cohorts (GSE8671). The data were presented as the mean ± SD of different samples. The significance was analyzed by the Student’s *t*-test. **P < 0.01, ***P < 0.001, ****P < 0.0001. **c** The correlation of FoxM1 and STMN1 expression in 31 solid tumors (including ACC, BLCA, BRCA, CESC, CHOL, COAD, ESCA, GBM, HNSC, KICH, KIRC, KIRP, LGG, LIHC, LUAD, LUSC, MESO, OV, PAAD, PCPG, PRAD, READ, SARC, SKCM, STAD, TGCT, THCA, THYM, UCEC, UCS and UVM) from the TCGA database was analyzed using the GEPIA platform, and the correlation of FoxM1 and STMN1 expression in LIHC (*n* = 373), GC (*n* = 415) and CRC (*n* = 328) TCGA data. **d** Protein levels of FoxM1 and STMN1 in LIHC (*n* = 79), GC (*n* = 61) and CRC (*n* = 68) were analyzed by IHC. The correlation of FoxM1 and STMN1 was analyzed by linear regression analysis

Since FoxM1 and STMN1 both play important roles in the process of cell cycle regulation, we wondered to know whether there is a functional link in cancers. To investigate the expression pattern of FoxM1 and STMN1 in different types of cancers, we first analyzed the mRNA levels of FoxM1 and STMN1 in the Oncomine database. The result showed that FoxM1 and STMN1 highly express in almost all types of solid tumors in a serial of independent cohorts (Fig. 1a), including gastric cancer, hepatocellular carcinoma and colorectal cancer (Fig. 1b). To figure out whether there is a general correlation between FoxM1 and STMN1 in cancers, we then analyzed the expression patterns of them. The results showed a significantly positive correlation between FoxM1 and STMN1 in all cancer samples derived from the TCGA database (Fig. 1c and Supplementary Table S1). To further confirm the relationship between these two molecules at the protein level, we performed immunohistochemistry (IHC) on the collected tissue chips to examine the expression pattern of FoxM1 and STMN1 in liver hepatocellular carcinoma (LIHC), gastric cancer (GC) and colorectal cancer (CRC) samples. The data showed a closely positive correlation between FoxM1 and STMN1 at the protein level (Fig. 1d and Supplementary Fig. S1). These results indicated that FoxM1 and STMN1 may have a positive regulatory relationship in multiple cancers.

### STMN1 is transcriptionally activated by FoxM1 in cancer cells

**Fig. 2:**
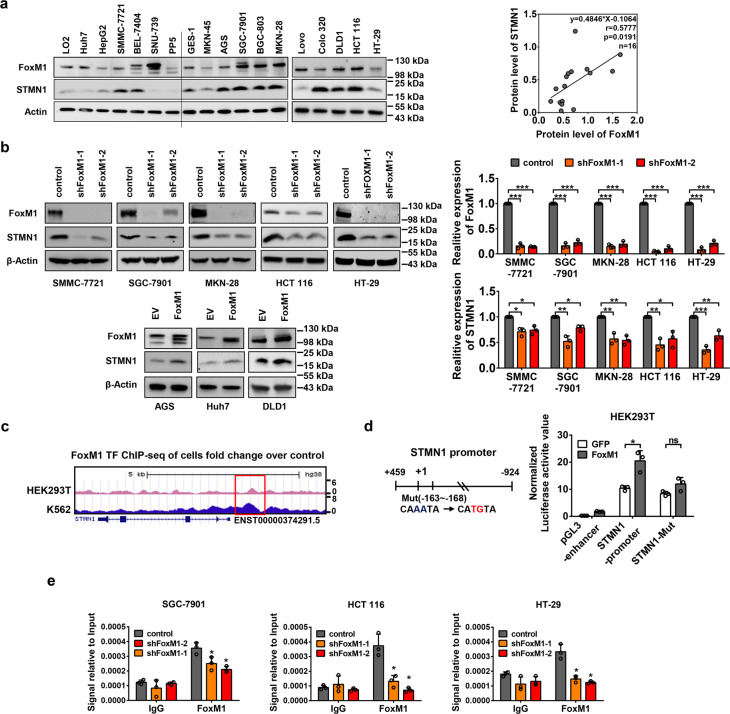
FoxM1 transcriptionally activates STMN1 in cancer cells. **a** The expression of FoxM1 and STMN1 in six hepatocellular carcinoma cell lines, five gastric cancer cell lines, five colorectal cancer cell lines, a human hepatocyte cell line LO2 and a human gastric mucosal epithelial cell line GES-1. The gray density analysis for the data of Western blot was done by using ImageJ software, and the correlation of FoxM1 and STMN1 expression was analyzed in the above 16 cell lines, excepting an LIHC cell line SNU-739 and a CRC cell line Colo-320. **b** The hepatocellular carcinoma cell line SMMC-7721, gastric cancer cell lines SGC-7901 and MKN-28, colorectal cancer cell lines HCT 116 and HT-29 were used to established FoxM1-knockdown cells by lentivirus-mediated shRNA delivery system. The human hepatocellular carcinoma cell line Huh7, gastric cancer cell line AGS and colorectal cancer DLD-1 were used to establish FoxM1-overexpressed cells by lentivirus infection. The protein levels of FoxM1 and STMN1 were detected using Western blot. The qRT-PCR assay was performed to detect the mRNA expression of STMN1 in FoxM1-knockdown or -overexpressed cells. The data were presented as the mean ± SD of three independent experiments. The significance was analyzed by the Student’s *t*-test. *P < 0.05, **P < 0.01, ***P < 0.001. **c** Analysis of the ChIP-seq data of FoxM1 in HEK293T and K562 cells from the ENCODE database, respectively. The red box shows the binding peaks of FoxM1 on the STMN1 genomic locus. **d** Dual luciferase reporter assay of FoxM1 and STMN1 promoter or its mutation. The binding site of FoxM1 is in the transcription start site of −163 ~ −168. The data were presented as the mean ± SD of three independent experiments. The significance was analyzed by the Student’s *t*-test. *P < 0.05. **e** The ChIP-qPCR was used to determine the direct binding of FoxM1 on the promoter region of STMN1 in SGC-7901, HCT 116 and HT-29 cell lines. Primers located on the upstream of STMN1 transcript start site (−2893 ~ −3000) was designed for a negative control. The data were presented as the mean ± SD of three independent experiments. The significance was analyzed by the Student’s *t*-test. *P < 0.05

To investigate the functional relationship between FoxM1 and STMN1 in cancers, we used Western blot to observe the expression pattern of FoxM1 and STMN1 in 18 cancer cell lines derived from LIHC, GC and CRC. Interestingly, we also noticed an unusual expression pattern of FoxM1 and STMN1 in two cell lines. In SNU-739, a liver cancer cell line, although there is a high level of FoxM1, the expression of STMN1 is very low, whereas in Colo-320 a CRC cell line, a low level of FoxM1 is accompanied with a high level of STMN1 (Fig. 2a). It may due to multiple regulatory mechanisms of STMN1 in these cells, such as epigenetic regulation, protein degradation or others. But there is a significant positive correlation of FoxM1 and STMN1 in most of the tested tumor cell lines (Fig. 2a). And then using two independent short hairpin RNAs (shRNAs), we knocked down endogenous FoxM1 and STMN1, respectively, in three different types of cancer cell lines. Strikingly, in tested five cancer cell lines, the protein levels of STMN1 dramatically reduced upon FoxM1 depletion (Fig. 2b), while we did not find obvious changes in FoxM1 protein level in STMN1-knockdown cells (Supplementary Fig. S2). We also performed gain-of-function experiments in FoxM1 lower expression cells and found that the protein levels of STMN1 increase with FoxM1 overexpression (Fig. 2b). These results indicated that STMN1 is a downstream gene regulated by FoxM1. FoxM1 has diverse ways to regulate gene expression. Among all mechanisms of FoxM1-mediated gene expression, transcriptional regulation is the most important way. To explore the mechanism by which FoxM1 regulates STMN1 expression, we performed RT-qPCR to test the mRNA levels of STMN1 in FoxM1-knockdown cancer cell lines. The data showed that loss of FoxM1 significantly decreases STMN1 expression in the mRNA level (Fig. 2b). This led us to hypothesize that FoxM1 may regulate STMN1 at the transcriptional level in cancer cells. We then analyzed the public-opened ChIP-seq database and found the obvious binding peaks of FoxM1 on the proximal region of STMN1 locus (Fig. 2c). To further validate the direct binding of FoxM1 on the promoter region of STMN1 in cancer cells, we performed dual luciferase reporter assay (Fig. 2d) and ChIP assay (Fig. 2e) in the above-mentioned LIHC, GC and CRC cancer cells. Because of the important role of FoxM1 for survival of SMMC-7721 (an LIHC cell line) and MKN-28 (a GC cell line), we even cannot obtain enough cells to perform the ChIP assay when FoxM1 was knocked down using the lentivirus-mediated shRNA. But in other three cell lines, the DNA fragments of STMN1 were pulled down using FoxM1 antibody. Our ChIP-qPCR data showed the physical binding of FoxM1 on the promoter region of STMN1, which is consistence with the bioinformatics analysis in the public ChIP-seq database. Taken together, we revealed a generally transcriptional regulation of FoxM1 on the expression of STMN1 in cancers.

### STMN1 promotes survival and proliferation of cancer cells in vitro

**Fig. 3:**
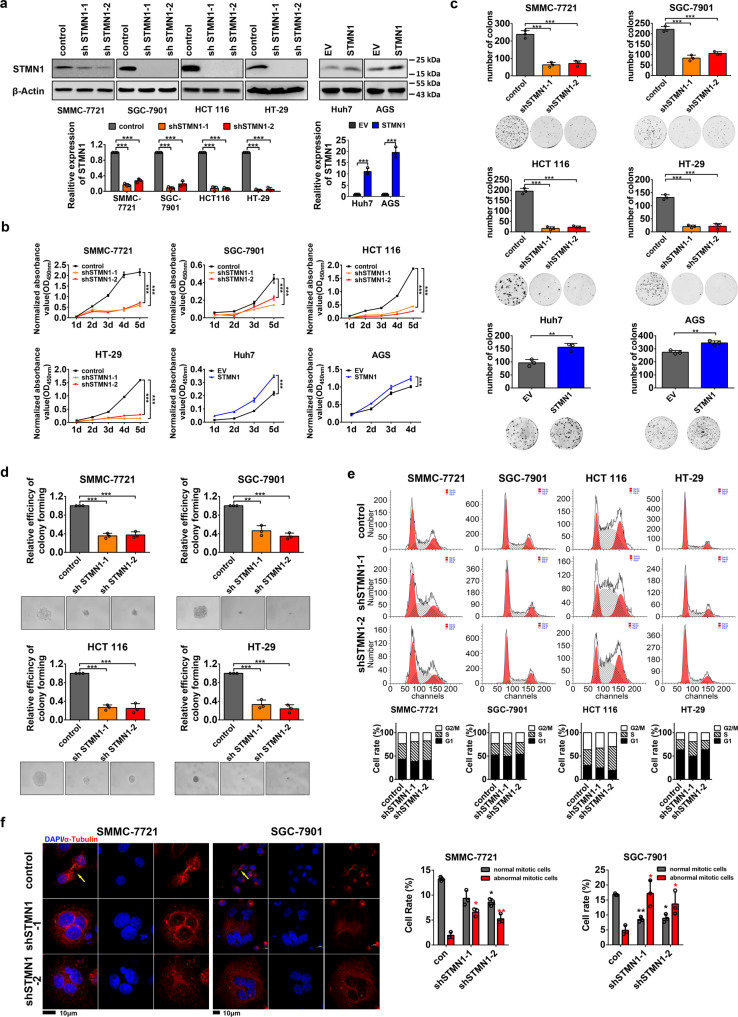
STMN1 is essential for survival and proliferation in cancer cells in vitro. **a** The hepatocellular carcinoma cell line SMMC-7721, gastric cancer cell line SGC-7901, colorectal cancer cell lines HCT 116 and HT-29 were used to establish STMN1-knockdown cells using pLKO.1 gene silence system. The hepatocellular carcinoma cell line Huh7, gastric cancer cell lines AGS were used to establish STMN1-overexpression cell lines by lentivirus infection. The protein levels of STMN1 were detected by Western blot, and the mRNA levels were detected by qRT-PCR. The data were presented as the mean ± SD of three independent experiments. The significance was analyzed by Student’s *t*-test. ***P < 0.001. **b** Cell viability of cells was detected by cell counting-8 kit (CCK-8). The data were presented as the mean ± SD of three independent experiments. The significance was analyzed by Student’s *t*-test. ***P < 0.001. **c** Plate clone assay was performed and the number of clones was measured by ImageJ software. The data were presented as the mean ± SD of three independent experiments. The significance was analyzed by Student’s *t*-test. **P < 0.01, ***P < 0.001. **d** Soft agar assay was performed and the tumorigenic sphere was photographed after 2 weeks. The data were presented as the mean ± SD of three independent experiments. The significance was analyzed by Student’s *t*-test. **P < 0.01, ***P < 0.001. **e** Cell cycle was analyzed by flow cytometry. **f** Cell mitosis was analyzed by immunofluorescence of α-Tubulin. The nuclei (blue) are stained with DAPI and the α-Tubulin (red) is stained with Alexa Flour 555. The images were captured at 60× magnification, and the scale bar is 10 μm. The yellow arrows point to the normal mitosis cells. The percentage of normal or abnormal cells was calculated by numbers of normal or abnormal nucleus divided by numbers of total nucleus × 100%. The data were presented as the mean ± SD of three different fields of view at low magnification (20×). The significance was analyzed by Student’s *t*-test. *P < 0.05, **P < 0.01

FoxM1 is a master regulator of cell division. Aberrantly high activation of its transcriptional activity functionally links to sustained survival and unlimited proliferation in transformed cells. Based on our finding that STMN1 is transcriptionally regulated by FoxM1, we wondered to know the biological function of STMN1 in cancer cells. We used two independent shRNAs to stably knock down STMN1 and test the survival and proliferation in cancer cells (Fig. 3a and Supplementary Fig. S3a). We also overexpressed STMN1 in Huh7 and AGS cells and tested survival and proliferation (Fig. 3a). We found that the cell viability of the cancer cells significantly reduces with depletion of STMN1 (Fig. 3b and Supplementary Fig. S3b). And the further clonogenic formation assay showed that in both 2D and 3D cell culture system, the number and the size of cell clones obviously decrease in STMN1-knockdown cells when compared with that in control group (Fig. 3c, d, Supplementary Fig. S3c, d). To investigate the influence of STMN1 on cell cycle and mitosis, we performed the immunofluorescent staining of α-Tubulin and the flow cytometry. The results showed that inhibition of STMN1 arrests cell cycle in S phase (Fig. 3e) and increases the percentage of abnormal mitosis cells, as formation of multinuclear cells (Fig. 3f). All data suggested that STMN1 plays a critical role in promoting tumor cell proliferation and sustaining cell cycle/mitosis in multiple types of cancer cells in vitro.

### Knockdown of STMN1 suppresses tumorigenesis in vivo

**Fig. 4:**
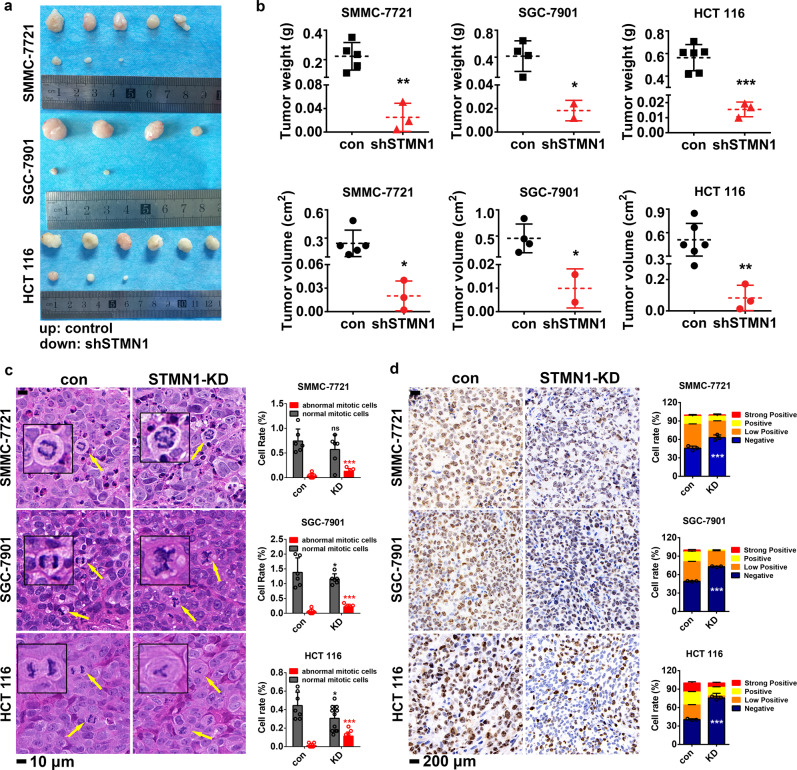
STMN1 affects tumorigenesis in vivo. The hepatocellular carcinoma cell line SMMC-7721, gastric cancer cell line SGC-7901 and colorectal cancer cell line HTC 116 infected with shSTMN1 lentivirus and control lentivirus were injected subcutaneously into the back of 6-year-old nude mice for 4–6 weeks to grow tumors. The left side of the mice was injected with control cells and the right side was injected with shSTMN1-expressing cells. **a** The mice were killed after 4–6 weeks and tumors were removed to measure the weight. The tumor volume was measured and calculated by *v* = 0.5 × Length × Width^2^. SMMC-7721, *n* = 5. SGC-7901, *n* = 4. HCT 116, *n* = 6. The data were presented as the mean ± SD. The significance was analyzed by Student’s *t*-test. *P < 0.05, **P < 0.01, ***P < 0.001. **c** HE staining was performed to observe the morphological changes of the nucleus. The yellow arrows show the typical normal or abnormal morphological changes of the nucleus in cell mitosis process. The scale bar is 10 μm. The percentage of normal or abnormal cells was calculated by numbers of normal or abnormal nucleus divided by numbers of total nucleus × 100%. The data were presented as the mean ± SD of 6–11 different fields of view at low magnification (20×). The significance was analyzed by Student’s *t*-test. *P < 0.05, ***P < 0.001. **d** Ki67 expression in the tumor was detected by IHC and the percentage of positive cells was calculated by ImageJ IHC Profiler. The scale bar is 200 μm. The data were presented as the mean ± SD of three different fields of view at low magnification (50×). The significance was analyzed by Student’s *t*-test. ***P < 0.001

To examine the oncogenic roles of STMN1 in vivo, we selected three cancer cell lines derived from LIHC, GC and CRC, respectively, to establish the xenograft model in nude mice. Cancer cells infected with STMN1-shRNA or pLKO.1-control lentivirus were subcutaneously injected into the BALB/c nude mice. It was shown that the tumor volume and tumor weight significantly decrease in the STMN1 deficiency groups (Fig. 4a, b), indicating that the STMN1-knockdown cells obviously lost the capacity of tumor growth in vivo. Moreover, pathological analysis of tumor tissue sections showed a much higher frequency of the occurrence of abnormal cell division in STMN1-knockdown groups (Fig. 4c). It further suggested that STMN1-induced tumorigenesis is related to cell mitosis. Additionally, the expression of Ki-67 reduces in cancer tissues with the depletion of STMN1 (Fig. 4d). All the above results demonstrated that STMN1 plays an oncogenic role in vivo in LIHC, GC and CRC cancers.

### STMN1 is essential for FoxM1-mediated proliferation of cancer cells

**Fig. 5:**
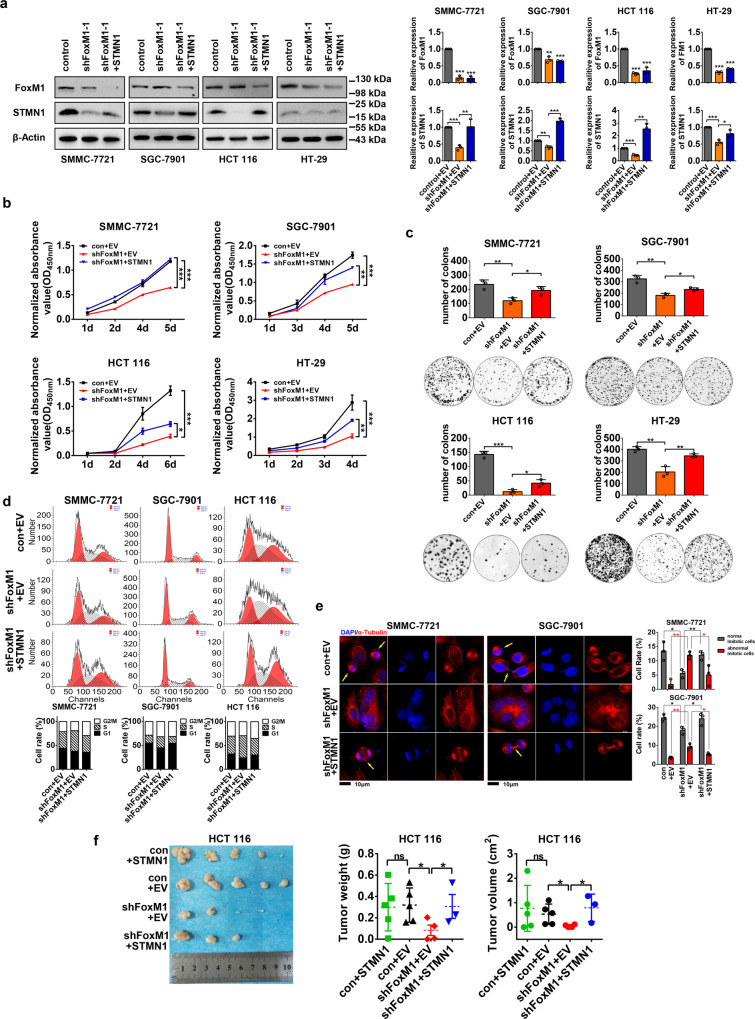
FoxM1-mediated cancer cell proliferation requires STMN1 expression. **a** The hepatocellular carcinoma cell line SMMC-7721, gastric cancer cell line SGC-7901, colorectal cancer cell lines HCT 116 and HT-29 were used to established FoxM1-silenced and STMN1-overexpressed cell lines by lentivirus-mediated system. The protein levels of FoxM1 and STMN1 were detected by Western blot and the mRNA levels were detected by RT-qPCR. The data were presented as the mean ± SD of three independent experiments. The significance was analyzed by Student’s *t*-test. *P < 0.05, **P < 0.01, ***P < 0.001. **b** Cell viability of cells was detected by cell counting-8 kit. The data were presented as the mean ± SD of three independent experiments. The significance was analyzed by Student’s *t*-test. *P < 0.05, **P < 0.01, ***P < 0.001. **c** Plate clone assay was performed and the number of clones was measured by ImageJ software. The data were presented as the mean ± SD of three independent experiments. The significance was analyzed by Student’s *t*-test. *P < 0.05, **P < 0.01, ***P < 0.001. **d** Cell cycle was analyzed by flow cytometry. **e** Cell mitosis was analyzed by immunofluorescence of α-Tubulin. The nuclei (blue) are stained with DAPI and the α-Tubulin (red) is stained with Alexa Flour 555. The images were captured at 60× magnification, and the scale bar is 10 μm. The yellow arrow points to the normal mitosis cells. The percentage of normal or abnormal cells was calculated by numbers of normal or abnormal nucleus divided by numbers of total nucleus × 100%. The data were presented as the mean ± SD of three different fields of view at low magnification (20×). The significance was analyzed by Student’s *t*-test. *P < 0.05, **P < 0.01. **f** Colorectal cancer cell lines HTC 116 were infected with lentivirus. The mice were separated into four groups, control group (cells infected with pLKO.1-control lentivirus and pLV-GFP lentivirus), FoxM1-knockdown group (cells infected with pLKO.1-shFoxM1 lentivirus and pLV-STMN1-GFPSpark lentivirus), rescue group (cells infected with pLKO.1-shFoxM1 lentivirus and pLV-STMN1-GFPSpark lentivirus) and STMN1-overexpressed group (cells infected with pLKO.1-control lentivirus and pLV--GFP lentivirus). Cells were injected subcutaneously into the back of 5-year-old nude mice for 4 weeks to grow tumors. The mice were killed and the tumors were removed to measure the weight and volume. Tumor volume was measured and calculated by *v* = 0.5 × Length × Width^2^; *n* = 5. The data were presented as the mean ± SD. The significance was analyzed by Student’s *t*-test. *P < 0.05

To investigate the role of STMN1 in FoxM1-mediated cell proliferation in cancer cells, we tested cell proliferation using five cancer cell lines derived from LIHC, GC and CRC, which were infected with control or shFoxM1 lentivirus along with empty vector (EV) or STMN1-overexpressed (STMN1) lentivirus. Western blot and RT-qPCR were performed to detect the expression of FoxM1 and STMN1 (Fig. 5a and Supplementary Fig. S4a). CCK-8 and plate colony assay were performed to test cell proliferation. We found that the cell viability decreases with FoxM1 depletion, and then raises up with STMN1-overexpression (Fig. 5b and Supplementary Fig. S4b). Further clonogenic formation assay also showed that the number of cell clones obviously decreases in FoxM1-knockdown cells and raises up with STMN1 overexpression (Fig. 5c and Supplementary Fig. S4c). We performed the flow cytometry and the immunofluorescent staining of α-Tubulin to test the contribution of a FoxM1–STMN1 axis on cell cycle/mitosis. It was showed that overexpression of STMN1 in FoxM1-knockdown cells partially rescues the cell cycle arrest (Fig. 5d) and mitosis (Fig. 5e), indicating that STMN1 can mediate functions of FoxM1 through impacting on cell cycle/mitosis. To further investigate the function of a FoxM1–STMN1 axis in vivo, we performed rescue experiments in nude mice. It was showed that overexpression of STMN1 in FoxM1-knockdown cells partially rescues the tumor growth in nude mice (Fig. 5f). We also established STMN1-overexpressed cell lines and performed in vivo experiments, but there was no obvious change in tumor weight and tumor volume compared with the control group (Fig. 5f). It can be explained that the endogenous levels of STMN1 in cancer cells have been enough for cell survival and tumorigenesis, the overexpression of STMN1 may be redundant. Collectively, these results demonstrated that STMN1 contributes to the FoxM1-induced proliferation and tumorigenesis of cancer cells in vitro and in vivo.

### High levels of FoxM1 and STMN1 are closely associated with poor prognosis in cancers

**Fig. 6:**
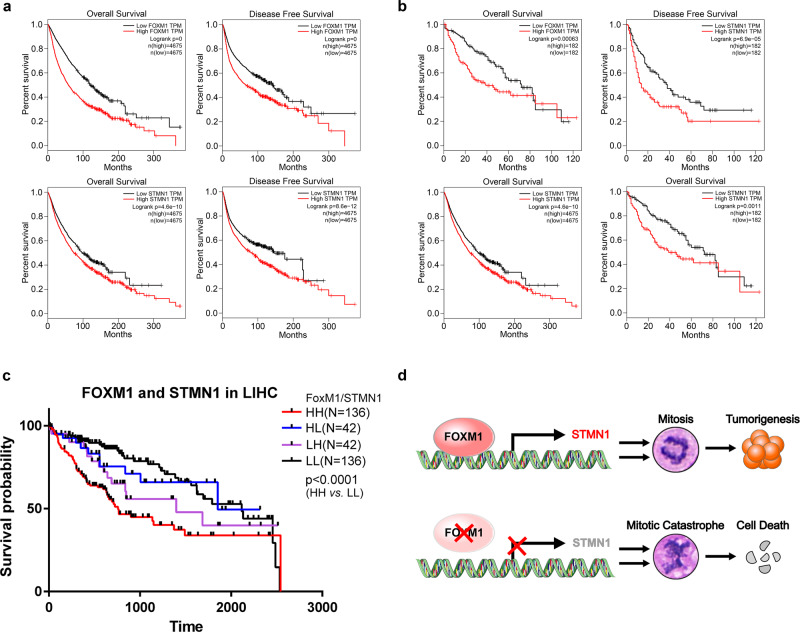
High activation of FoxM1–STMN1 signal axis is a risk of poor prognosis in cancers. **a** Overall survival and disease-free survival rate of 31 solid tumors (including ACC, BLCA, BRCA, CESC, CHOL, COAD, ESCA, GBM, HNSC, KICH, KIRC, KIRP, LGG, LIHC, LUAD, LUSC, MESO, OV, PAAD, PCPG, PRAD, READ, SARC, SKCM, STAD, TGCT, THCA, THYM, UCEC, UCS and UVM) from TCGA database were analyzed using the GEPIA platform; *n* = 9350. **b** Overall survival and disease-free survival rate of LIHC from TCGA database were analyzed using the GEPIA platform; *n* = 364. **c** Overall survival rate of LIHC from the TCGA database was analyzed according to the mRNA levels of FoxM1 and STMN1. HH, FoxM1^High^/STMN1^High^ (*n* = 136). HL, FoxM1^High^/STMN1^Low^ (*n* = 42). LH, FoxM1^Low^/STMN1^High^ (*n* = 42). LL, FoxM1^Low^/STMN1^High^ (*n* = 136). **d** A working model showing that aberrantly high activation of FoxM1–STMN1 axis contributes to tumorigenesis in cancer cells by promoting cell mitosis, while interruption of FoxM1–STMN1 axis can result in mitotic catastrophe-mediated cell death

To investigate the effect of FoxM1 and STMN1 on cancer progression, we performed survival analysis of all solid tumors from TCGA database according to FoxM1 and STMN1 expression. The result showed that patients bearing high levels of FoxM1 and STMN1 solid tumors have lower overall survival rate and disease-free survival rate (Fig. 6a). To further confirm this effect in specific types of cancers, we analyzed the survival of LIHC and STAD from TCGA database. The results showed a positive correlation of high levels of FoxM1 or STMN1 with poor prognosis of LIHC (Fig. 6b). To further investigate whether the co-overexpression of FoxM1 and STMN1 has an effect on LIHC progression, we analyzed the expression status of STMN1 and FoxM1. The LIHC patients were divided into four groups (Fig. 6c): (1) FoxM1^High^/STMN1^High^ (*n* = 136), (2) FoxM1^High^/STMN1^Low^ (*n* = 42), (3) FoxM1^Low^/STMN1^High^ (*n* = 42), (4) FoxM1^Low^/STMN1^Low^ (*n* = 136). The result showed that patients bearing FoxM1^High^/STMN1^High^ teratocarcinomas have high significantly shorter overall survival (*P* < 0.01) than patients whose tumors overexpressed either one or neither of the molecules (Fig. 6c). In addition, we also performed survival analysis using Kaplan–Meier Plotter. The results showed that high levels of FoxM1 and STMN1 also exhibit a poor prognosis of GC patients, though it showed no significance (*P* = 0.081) (Supplementary Fig. S4). The data indicated that high levels of FoxM1 and STMN1 are closely associated with poor prognosis in cancers, thus shedding light on the prognostic value of combined utilization of FoxM1 and STMN1.

## Discussion

### The biological function of FoxM1 in the cancer-promoting signaling networks

FoxM1 has been found with aberrantly high expression in almost all kinds of cancers, including lung cancer, glioblastoma, prostate cancer, basal cell carcinoma, hepatocellular carcinoma, breast cancer and primary pancreatic cancer.^2,3^ Our analysis in Oncomine and tissue arrays also showed that the FoxM1 expression is abnormal in hepatocellular carcinoma, gastric cancer and colorectal cancer (Fig. 1b, d). FoxM1 is reported as an important effector in response to several oncogenic signal pathways and also facilitates in cell growth, angiogenesis, tumor invasion, DNA damage repair, senescence and cell cycle.

FoxM1 is reported frequently overexpressed in many kinds of cancer types and contributing to promote cell cycle and leads to excessive cell growth. The main function of FoxM1 is as a transcriptional factor to promote cell cycle-related gene expression. Moreover, FoxM1 also can cross-talk with other signal pathways and promote progression of cancers. For example, FoxM1 is an effector of Raf/MEK/MAPK in G2/M phase, and on the other hand, activation of Raf/MEK/MAPK pathway can enhance the activation of FoxM1 and cyclinB1.^15^ In the vascular endothelial growth factor (VEGF) signaling pathway, it was reported that FoxM1 directly binds with the promoter region of VEGF gene, and consequently promotes angiogenesis, migration and invasion of tumor cells.^16,17^ In the matrix metalloproteinase (MMP) signaling pathway, FoxM1 can regulate MMPs and promote tumor cell invasion.^18^ Overexpression of FoxM1 can significantly inhibit senescence and the expression of p53 and p21^cip1^.^19^ In Wnt/β-Catenin signal pathway, FoxM1 protein interacts with β-Catenin and induces the nuclear translocation of β-Catenin in both CML cells and glioma cells.^20,21^ In breast cancer cells, direct interaction between FoxM1 and Smad3 has been confirmed and it induces transcription of TGF-β/Smad3-mediated target genes.^9^ FoxM1 acts as a protective partner of Smad3 protecting Smad4 from degradation, inducing TGF-β signal transduction and promoting breast cancer metastasis.^9^ FoxM1 can also interact with YAP/TEAD complex and regulate the expression of CIN-associated genes, then results in genomic instability in LIHC.^10^ And a recent study showed that alter N6-methyladenosine (m6A) RNA modification of FoxM1 by ALKBH5 and a long non-coding RNA antisense FoxM1 in glioblastoma enhance transcription of FoxM1 and proliferation of patient-derived GSCs.^22^ Other studies also showed that FoxM1-mediated pathways induce resistance of some anticancer drugs. An Akt/FoxM1/STMN1 pathway was found as a mechanism for tyrosine kinase inhibitors (TKIs) resistance in non-small cell lung cancer (NSCLC).^15^ Since a growing body of evidence has demonstrated the importance of FoxM1 as a hub gene in the oncogenic network, it is emergent to further identify the novel downstream targets regulated by FoxM1. The meaning of our study is to provide more potential targets for the treatment of FoxM1-driven cancers.

### STMN1 regulatory network plays vital roles in cell cycle and mitosis

STMN1 gene encodes a protein involved in the regulation of the microtubule filament system by destabilizing microtubules.^23^ It is essential for cell cycle and mitosis. Inhibition of STMN1 results in reduction of cellular proliferation and accumulation of cells in G2/M.^11^ In the human fibroblasts, deletion of STMN1 induces genomic instability during mitosis and consequently leads to senescence.^24^ In tumor cells, STMN1 is a tumor promoter contributing to cell proliferation,^25^ invasion, migration^26,27^ and drug resistance.^14,28^ Several studies showed that inhibition of STMN1 can promote the sensitivity of anti-mitotic agents such as taxanes (i.e., docetaxel) and vinca alkaloids (i.e., vinblastine, vincristine). Similarly, STMN1 silencing significantly reduces proliferation and induced-apoptosis in response to ruxolitinib.^29^ It was also reported that siRNA-based inhibition of STMN1 increases the sensitivity of colorectal cancer cells to the treatment of 5-FU.^30^ Moreover, the inhibition of STMN1 by the anti-cancer potency of three novel indoly-chalcones (CITs) can induce mitotic catastrophe in pancreatic cancer.^31^

Abnormally high expression of STMN1 is seen in many kinds of cancers, but the mechanisms of the dysregulated expression are inadequate. Previous studies have shown that the regulation of STMN1 in cell mitosis mainly depends on post-transcriptional regulation. On the one hand, phosphorylation of Ser16, Ser25, Ser38 and Ser63 of STMN1 can be catalyzed by protein kinase like CAM II,^32^ CDK1, CDK2,^33^ MAPK and Kinase downstream of TNF, which control the formation of the mitotic spindle and progression of mitosis by maintaining the dynamic balance of microtubules.^34^ Phosphorylated STMN1 promotes continuous assembly of microtubules and formation of mitotic spindle in G2/M phase. Ultimately it enables the cell cycle process to enter into mitosis successfully. While in the later stage of mitosis, STMN1 is dephosphorylated by protein phosphatase, and the non-phosphorylated STMN1 promotes depolymerization of microtubules and the disassembly of mitotic spindle, which then contributes to complete spindle division and exit from mitosis.^11^ Consequently, the phosphorylation status of STMN1 determines not only whether the cells can enter into mitosis, but also whether the cell cycle can timely exit from mitosis and enter into cytoplasmic division at the later stage of mitosis. On the other hand, it has been reported that STMN1 is post-transcriptionally regulated by miRNAs, such as miR-101,^35^ miR-34a,^36,37^ miR-193b^38^ miR-493 (ref. ^39^) and miR-223.^40,41^ STMN1 is inhibited by miR-34a and induces expression of growth differentiation factor 15 (GDF15) in prostate cancer cells, which finally promotes cell proliferation and invasion.^37^ In our previous study, we also demonstrated that STMN1 is a direct target of tumor suppressive miR-101 in LIHC cells.^42^

In TGGA data analysis, we found that the expression of STMN1 in tumor tissues is also higher than that in adjacent tissues, which indicated that the oncogenic roles of STMN1 not only depends on post-transcriptional regulation, but also depends on the mRNA level. But the transcriptional regulation of STMN1 is rarely studied. Our study showed that FoxM1 upregulates STMN1 by transcriptional regulation, and the FoxM1-mediated transcriptional activation of STMN1 can promote cell proliferation and survival both in vitro and in vivo in LIHC, GC and CRC cancers. It revealed that a FoxM1–STMN1 axis may be a general regulatory mechanism of STMN1 in solid tumors.

### A FoxM1–STMN1 axis facilities tumor growth by promoting cell cycle progression in solid tumors

In this study, we demonstrated that a general FoxM1–STMN1 regulatory axis contributes to cell proliferation and tumorigenesis in cancers. FoxM1 is aberrantly high-expressed in almost all solid cancers in our data analysis, like bladder cancer, breast cancer, sarcoma, colorectal cancer and lung cancer (Fig. 1a). Furthermore, the high-expressed FoxM1 is associated with poor prognosis, so this molecule is a biomarker of malignancy of tumor. We found that the mRNA levels of FoxM1 and STMN1 in transformed tissues are higher than those in adjacent tissues of multiple cancer (Fig. 1a). Then we found that the expression pattern of the two molecules has a remarkable positive correlation in most malignant tumors (Fig. 1c and Supplementary Table 1). We then tested the expression pattern of FoxM1 and STMN1 in 18 cancer cell lines derived from LIHC, GC and CRC, and found a significantly positive correlation between FoxM1 and STMN1 in tumor cells (Fig. 2a). It revealed that there may exist a general regulatory relationship between FoxM1 and STMN1 in solid tumor cells. To confirm the regulation between FoxM1 and STMN1, we established FoxM1 or STMN1 knockdown cell lines and found that FoxM1 can upregulate STMN1. The cancerous characteristic of FoxM1 mainly owes to its transcriptional activity. As a transcription factor, it can bind with conservative DNA sequences “AT/CAAAT/CA” and then activate gene expression. Our previous study also showed that silencing of FoxM1 in human LIHC cells results in cell cycle arrest and inhibits cell survival depending on downregulated CCNB1 in G2/M.^4^ In our present study, our luciferase report assay, ChIP-qPCR and ChIP-seq data analysis in public database demonstrate the mechanism that STMN1 is a direct target of FoxM1 in LIHC, GC and CRC (Fig. 2c–e). And the FoxM1–STMN1 axis promotes cell proliferation and tumorigenesis by maintaining cell cycle/mitosis (Fig. 5d, e). To confirm its effect on tumor progression, we performed survival analysis of LIHC patients. Surprisingly, we found that not only patients bearing FoxM1^high^ or STMN1^high^ tumors have a low survival, but patients bearing FoxM1^high^/STMN1^high^ tumors have the highest risk of poor prognosis, as the data shown in Fig. 6c. It indicates that LIHC overexpressing both FoxM1 and STMN1 is more aggressive, thus shedding light on the prognostic role of combined use of FoxM1 and STMN1. Though the positive correlation between high STMN1 expression and poor prognosis of GC and CRC patients is not as apparently as that in LIHC, there is a trend that GC or CRC patients bearing highly STMN1-expressed tumor have poor prognosis (Supplementary Fig. S5). It may because that dynamic changes in the phosphorylation level of STMN1 is the main event of mitotic process besides the transcription of mRNA. Another reason might be the drug resistance of high STMN1 expression patients.

In conclusion, we demonstrated that a general regulatory FoxM1–STMN1 axis promotes cell proliferation and tumorigenesis in FoxM1-driven cancers in vitro and in vivo (Fig. 6d). And survival analysis showed that the FoxM1–STMN1 axis promotes tumor progression. Our results revealed that the combination of the two molecules can be a more precise biomarker for prognostic prediction and a potential cancer treatment strategy.

## Methods and materials

### Materials

Rabbit monoclonal antibodies against FoxM1 (5436S; 1:1000) and STMN1 (13655S; 1:1000) for WB were obtained from Cell Signaling Technology (Cell Signaling Technology, Beverly, MA, USA). Mouse monoclonal antibodies against FoxM1 for IHC (sc-376471; 1:50) and ChIP assay were purchased from Santa Cruz Biotechnology (Dallas, TX, USA). Mouse monoclonal antibody against β-actin (A5316; 1:2000) was purchased from Sigma-Aldrich (St. Louis, MO, USA). Rabbit polyclonal antibody against α-Tubulin (11224-1-AP; 1:200) for immunofluorescence was purchased from Proteintech (Wuhan, Hubei, China). Horseradish peroxidase-conjugated goat anti-mouse and anti-rabbit secondary antibodies were obtained from Thermo Fisher Scientific. Lipofectamine Reagent 2000 transfection reagent was obtained from Invitrogen (Waltham, MA, USA).

### Cell lines and cell culture conditions

Human hepatocellular carcinoma cell line SMMC-7721, human gastric cancer cell lines SGC-7901 and MKN-28 were cultured in PRMI Medium 1640 (GIBCO BRL, Grand Island, NY, USA) with 10% fetal bovine serum (GIBCO BRL), penicillin (100 mg/ml) and streptomycin (100 mg/ml). Human colon cancer cell lines HT-29 were cultured in PRMI Medium 1640 with 20% fetal bovine serum. Human hepatocellular carcinoma cell line Huh7, human gastric adenocarcinoma cell line AGS, human colon cancer cell line HCT 116 and human embryonic renal epithelial cell line HEK293T were cultured in Dulbecco’s Modified Eagle Medium (GIBCO BRL) with 10% fetal bovine serum (GIBCO BRL), penicillin (100 mg/ml) and streptomycin (100 mg/ml). Human hepatocellular carcinoma cell line Huh7 and SMMC-7721 were obtained from the Type Culture Collection of the Chinese Academy of Sciences. Human gastric cancer cell lines SGC-7901 and MKN-28 were purchased from Genechem (Shanghai, China). Human gastric cancer cell line AGS, human colon cancer cell lines HT-29, HCT 116 and human embryonic renal epithelial cell line HEK293T cell line was purchased from ATCC (Manassas, VA, USA).

### DNA construction

The human FoxM1 shRNA was constructed by ligation of oligonucleotide sequences targeting human FoxM1 and STMN1 into the Age I and EcoR I digested pLKO.1-TRC cloning vector (Addgene, Cambridge, MA, USA; 10878). The sequences of shRNAs are listed in Supplementary Table S2. The pCDH-STMN1 plasmid was constructed via insertion of a PCR-amplified human STMN1 cDNA into a pCDH vector digested with BamH I and EcoR I. The pLV-STMN1-GFPSpark plasmid was purchased from Sino Biological (Beijing, China; HG15440-ACGLN).

### Lentivirus packaging and infecting

To generate FoxM1/STMN1-knockdown cell lines SMMC-7721, SGC-7901, MKN-28, HT-29 and HCT 116, a lentiviral pLKO.1-shRNAs plasmid targeting FoxM1/STMN1 was constructed. Lentiviruses were produced by con-transfecting pLKO.1-shFoxM1/pLKO.1-shSTMN1 plasmids and packaging plasmids (psPAX2 and pMD2.G) into HEK293T cells. Cancer cells were infected by the lentivirus and selected by 2 µg/ml puromycine for 3 days. To generate STMN1-overexpressed cell lines, a lentiviral pCDH-STMN1 plasmid expressing STMN1 was constructed and a pLV-STMN1-GFPSpark plasmid purchased from Sino Biological was used. Lentivirus was produced by contransfecting with pCDH-STMN1/pCDH/pLV-STMN1-GFPSpark/pLV-GFP plasmid and packaging plasmids (psPAX2 and pMD2.G) into HEK293T cells. For rescue experiments, the cells were infected with pLKO.1-shFoxM1/pLKO.1-control along with pCDH-STMN1/pCDH/pLV-STMN1-GFPSpark/pLV-GFP lentivirus and selected by 2 µg/ml puromycine for 3 days.

### Western blot

Cells were collected and washed with phosphate buffer saline (PBS) three times, and then harvested using RIPA Lysis Buffer. Proteins in the cell lysate were resolved on 10–15% SDS-polyacrylamide gel and transferred to a nitrocellulose membrane. Before incubation with primary antibodies, the membrane was blocked with 5% non-fat milk. Membranes were incubated with primary antibodies against FoxM1, STMN1 and β-Actin overnight at 4 °C. After incubation with peroxidase-conjugated secondary antibodies for an hour at room temperature, the signals were visualized using ECL chemiluminescent regents by Tanon 5500 (Tanon Science & Technology; Shanghai, China).

### Quantitative RT-PCR

RNA isolation and quantitative real-time RT-PCR were performed as previous described. Briefly, 5 × 10^6^ cells were harvested for purification of total RNA using TRIzol Reagent (Invitrogen), and 1 μg of total RNA of each sample was reversed to cDNA by PrimeScript RT Master Mix (TaKaRa, Tokyo, Japan). For detecting the mRNA levels of specific genes, the diluted cDNA of each sample was used as a template to perform quantitative PCR and the amplifications were done using SYBR-green PCR MasterMix (TaKaRa). PCR assays were performed three times and the fold changes of genes were obtained after normalizing to β-Actin using the comparative Ct method (fold change = 2^−ΔΔCt^). Primers used for quantitative RT-PCR are listed in Supplementary Table S3.

### Dual luciferase reporter assay

Cells were co-transfected with pGL3-STMN1-promoter/mutation, pCMV-FoxM1 and pRL-TK using Lipofectamine 2000 (Invitrogen). Cell extracts were prepared and luciferase activity was measured using the Dual Luciferase Reporter Assay System (Promega, Madison, WI, USA). The relative firefly luciferase activity was normalized with its respective Renilla luciferase activity.

### Chromatin immunoprecipitation assay

The chromatin immunoprecipitation analysis was performed as described previously using a SimpleChIP Enzymatic Chromatin IP Kit (Cell Signaling Technology, 9003). Gastric cancer cell line SGC-7901, colon cancer cell lines HCT 116 and HT-29 were infected with control or two shFoxM1 lentiviruses. A total of 1.2 × 10^7^ cells were crosslinked with 1% formaldehyde solution for 15 min at room temperature. The crosslink reaction was then stopped by addition of 10% glycine and lysed in 1 ml lysis buffer on ice. Lysates was harvested and sonicated into DNA fragments with 150–900 bp using the *Micrococcal* nuclease and Scientz-1500F Ultrasonic disperser (Ning Bo, China). Sonicated samples were spun down and subjected to overnight immunoprecipitation with IgG or FoxM1 antibody (Santa Cruz Biotechnology). After the proteins and RNA are removed by Protease K and RNAase A, the chromatin pulled-down by antibodies is purified. The enrichment of STMN1 is detected by qPCR amplification. Primers for qPCR amplification are listed in Supplementary Table S4.

### Cell cycle assay

Cells were infected with lentivirus and harvested by trypsinization and centrifugation. Cells were then fixed in 75% ethanal overnight at −20 °C. Then cells were stained with 10 µg/ml PI in PBS plus RNase. Then the cells were analyzed by a flow cytometer.

### α-Tubulin staining

Cells were fixed with 4% paraformaldehyde for 10 min, permeabilized with 0.1% Triton X-100 for 20 min, blocked with 5% BSA for 30 min and labeled with α-Tubulin antibody (Proteintech, Wuhan, Hubei, China, 11224-1-AP; 1:200) overnight at 4 °C, and then stained with Alexa Fluor 555 (Life Technologies, A21428). The nuclei were stained with DAPI. Images were visualized using a Nikon confocal microscope.

### Cell viability assay

Cell viability was analyzed using Cell Counting Kit-8 Kits. The cells were pre-seeded in 96-well plates with the number of 1 × 10^3^. The cell culture medium was discarded and replaced with culture medium containing 0.05 µg/µl Cell Counting Kit-8 (0.5 mg/ml) reagent and cells were incubated at 37 °C. After 0.5–4 h, the absorbance of the culture medium was detected using a Bio-RAD (Hercules, CA, USA) Microplate Reader with a wavelength of 450 nm. This procedure was repeated every day in the following 4–5 days.

### Colony formation assay

Long-term cell survival was monitored in a colony formation assay. In brief, 1000 cells were seeded into 6-well plates and allowed to grow for 2 weeks. The cells were fixed with 4% paraformaldehyde for 15 min and visualized by 0.5% (w/v) crystal violet (Sigma-Aldrich) staining. Colons in the plate were scanned using Odyssey Scanner (LI-COR, Lincoln, NE, USA) and the number of colons was quantified by Image J software.

### Soft agar assay

The cell survival in 3D culture was monitored by soft agar assay. Cells were plated in six-well plates with the bottom layer containing 0.5% low-melting agarose. Cells (3000–5000 per well) were mixed with low-melting agarose to a final concentration of 0.3% and layered over the bottom agar. The dishes were then cultured at 37 °C for 2–3 weeks and 500 μl of the culture medium was added to keep the top layer moist. Spheres were photographed by a digital camera coupled to a microscope.

### Xenograft experiment

To generate mouse subcutaneous tumors, SGC-7901, SMMC-7721 and HCT 116 cells were infected with control lentivirus or shSTMN1 lentivirus. Male 5- to 6-week-old BALB/c nude mice were implanted subcutaneously in the flank of back with 5 × 10^6^ SGC-7901 GC cells, SMMC-7721 LIHC cells and HCT 116 CRC cells. The mice were killed after 4–5 weeks and in vivo solid tumors were dissected and weighed. For rescue experiments, HCT 116 cells were infected with control/shFoxM1 lentivirus and pLV-GFP/pLV-STMN1-GFPSpark lentivirus. Male 5-week-old BALB/c nude mice were implanted subcutaneously in the flank of back with 6 × 10^6^ cells. The mice were killed after 4 weeks and in vivo solid tumors were dissected and weighed. The tumor volume was determined using the formula 0.5 × *L* × *W*^2^, where *L* is the longest diameter and *W* is the shortest diameter. The tumors were removed into 4% polyformaldehyde solution for fixing tissues.

### Hematoxylin and eosin staining

Hematoxylin and eosin (HE) staining was performed according to a conventional method. In short, tissues were fixed in 10% neutral buffered formalin and were embedded in paraffin and processed by standard histological techniques. Sections were placed in CAT hematoxylin for 1 min followed by rinsing in tap water to blue. The slides were stained with eosin solution for 3 min, dehydrated with graded alcohol, and washed with xylene. The slides were then photographed using fluorescence microscope. Quantitation of mitotic cells/total cell number was done on six of 20× fields of view from 2 or 3 mice.

### Immunohistochemistry

Slides of paraffin-embedded tumor tissue were deparaffinized and antigen repaired before blocking endogenous peroxidase with 3% hydrogen peroxide for 20 min. The tissue slides were treated with 0.01 mol/l sodium citrate (pH 6.0) in a microwave oven for 10 min after deparaffination and rehydration. The sections were then incubated with anti-Ki67 antibodies above at 4 °C overnight, subsequently developed using the Peroxidase/DAB, Rabbit/Mouse. The slides were then counterstained with hematoxylin. The ImageJ IHC profiler was used to quantify Ki67-positive cells number on three of 40× fields of view from 2 or 3 mice.

### Tissue microarray assay

Tissue microarrays of human LIHC, GC and CRC were obtained from the Department of Pathology, Fourth Military Medical University (China, Xi’an), and was stained with anti-human FoxM1 (Santa Cruz Biotechnology; sc-376471; 1:50) and STMN1 (Cell Signaling Technology, 13655S; 1:100) antibodies. The slides were scanned using Pannoramic (Santa Clara, CA, USA) MIDI and quantified using Quant center. The correlation of protein expression was analyzed using GraphPad Prism (Version 6; La Jolla, CA, USA).

### Public database

The expressions of FoxM1 and STMN1 in different cancer tissues and normal tissues were analyzed using Oncomine database (https://www.oncomine.org/). The mRNA levels of FoxM1 and STMN1 in hepatocellular carcinoma, gastric cancer and colorectal cancer patients were obtained from Gene Expression Omnibus (GEO, https://www.ncbi.nlm.nih.gov/geo/) and normalized using with GEO2R. Correlation analysis of FoxM1 and STMN1 from 31 TCGA solid tumors was done using Gene Expression Profiling Interactive Analysis (GEPIA, http://gepia.cancer-pku.cn). Oncoprint and correlation data of FoxM1 and STMN1 from TCGA were obtained by using the cBioportal for cancer genomics (http://www.cbioportal.org). Survival analysis of 31 solid tumors and LIHC from TCGA data was performed using Gene Expression Profiling Interactive Analysis (GEPIA, http://gepia.cancer-pku.cn). Kaplan–Meier Plotter (http://kmplot.com/analysed/) was used to perform survival analysis of GC.

### Statistics

The in vitro experiments were repeated at least three times unless stated otherwise. As indicated in the figure legends, all quantitative data are presented as the mean ± SD of three biologically independent experiments or samples. Statistical analysis was performed using GraphPad Prism 6. Statistical significance was tested using a two-tailed unpaired or paired Student’s *t*-test. The correlation of protein expression of FoxM1 and STMN1 was analyzed by linear regression analysis. The Kaplan–Meier survival was analyzed by Log-rank (Mantel-Cox) test analysis. A *P* value lower than 0.05 was considered significant.

## Supplementary information

Supplementary Materials

Supplementary Figure S1

Supplementary Figure S2

Supplementary Figure S3

Supplementary Figure S4

Supplementary Figure S5

## Data Availability

All data supported the paper are present in the paper and/or the Supplementary Materials. The original datasets are also available from the corresponding author upon request.
