# Supplementary figures and images for: Aberrantly high activation of a FoxM1–STMN1 axis contributes to progression and tumorigenesis in FoxM1-driven cancers
Source: Signal Transduct Target Ther. 2021 Feb 1;6:42. doi: 10.1038/s41392-020-00396-0 (PMC7851151; doi:10.1038/s41392-020-00396-0)

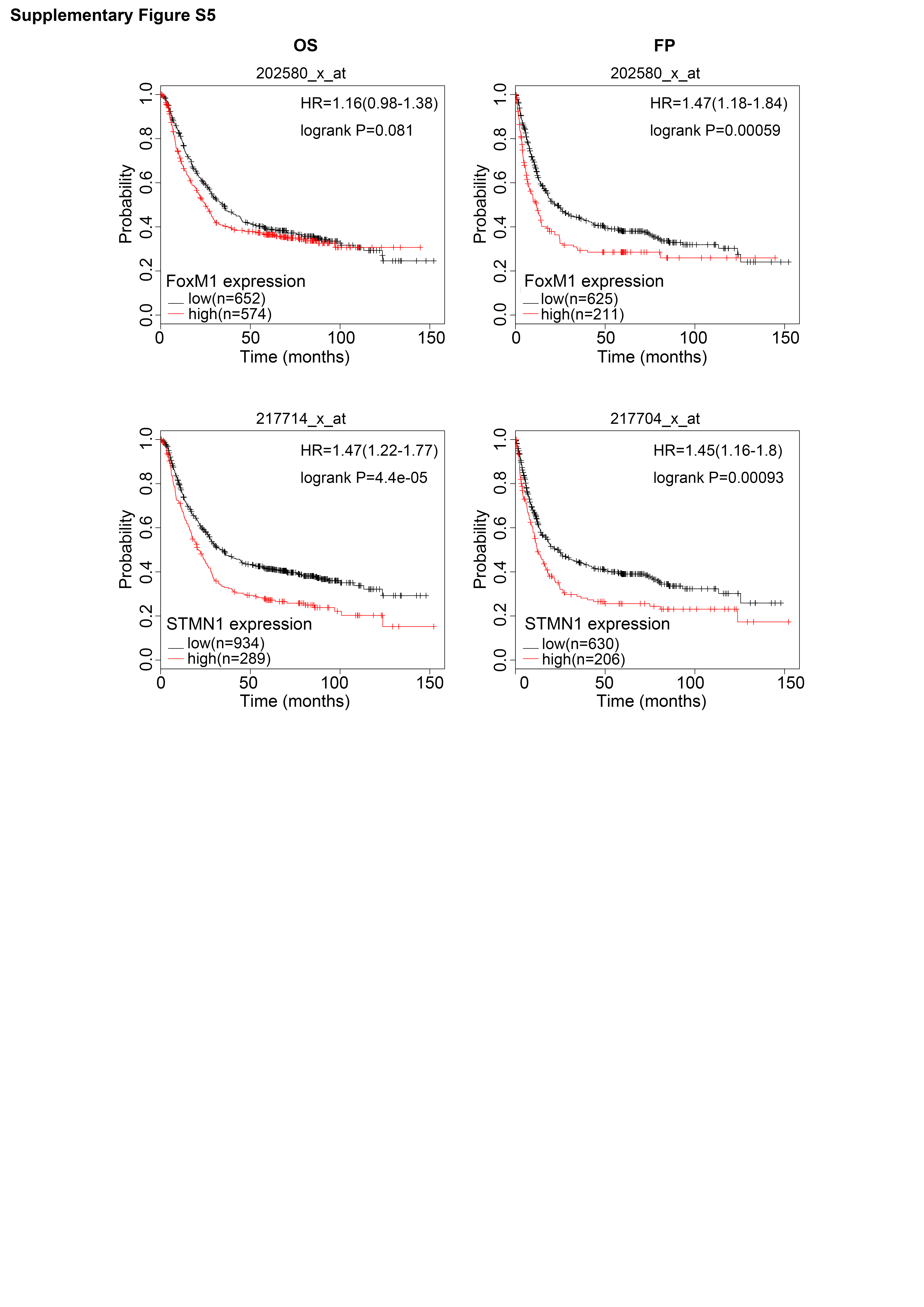

Supplement: Supplementary file 1 — Supplementary Materials [file 41392_2020_396_MOESM1_ESM.tif]

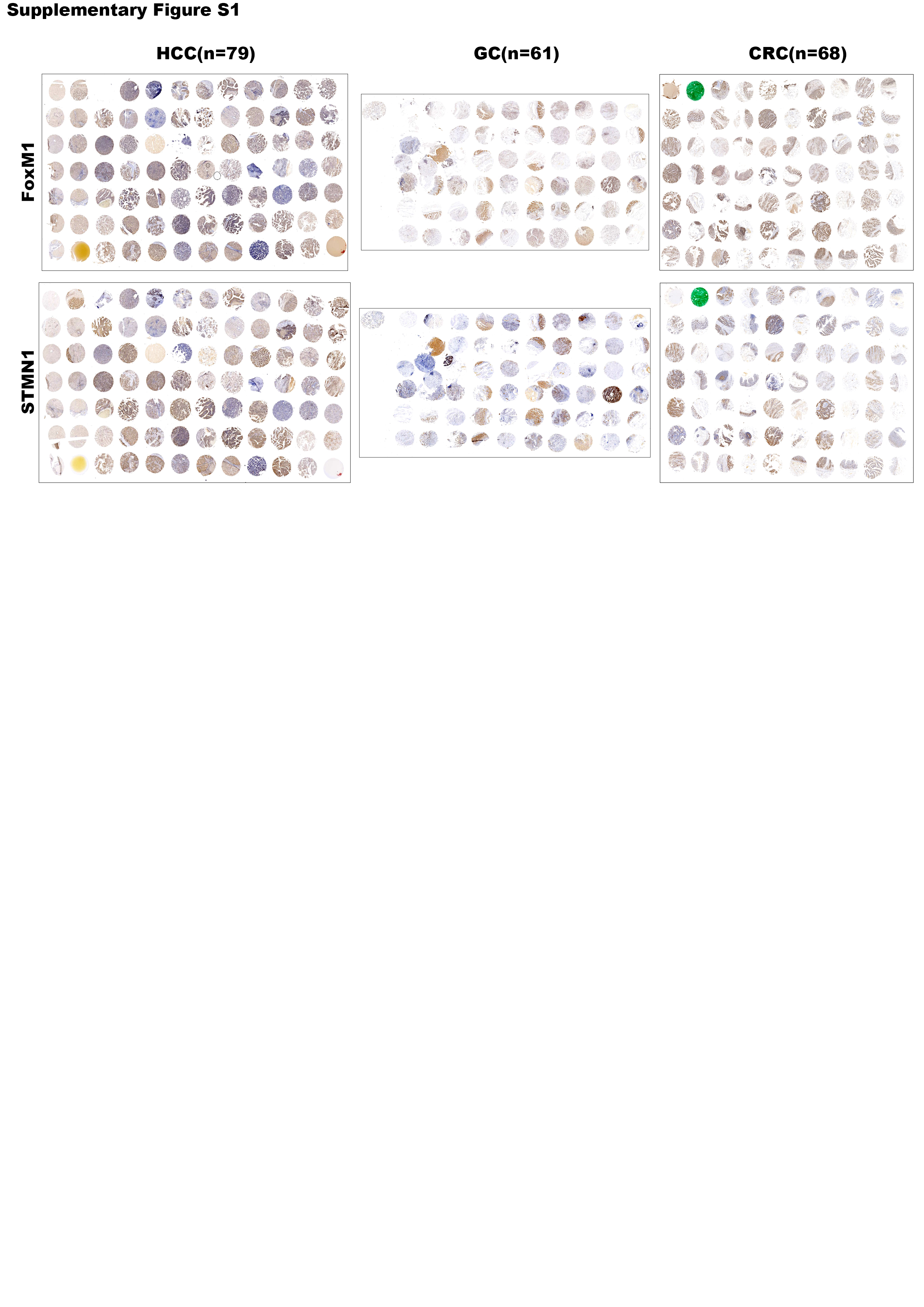

Supplement: Supplementary file 3 — Supplementary Figure S2 [file 41392_2020_396_MOESM3_ESM.tif]

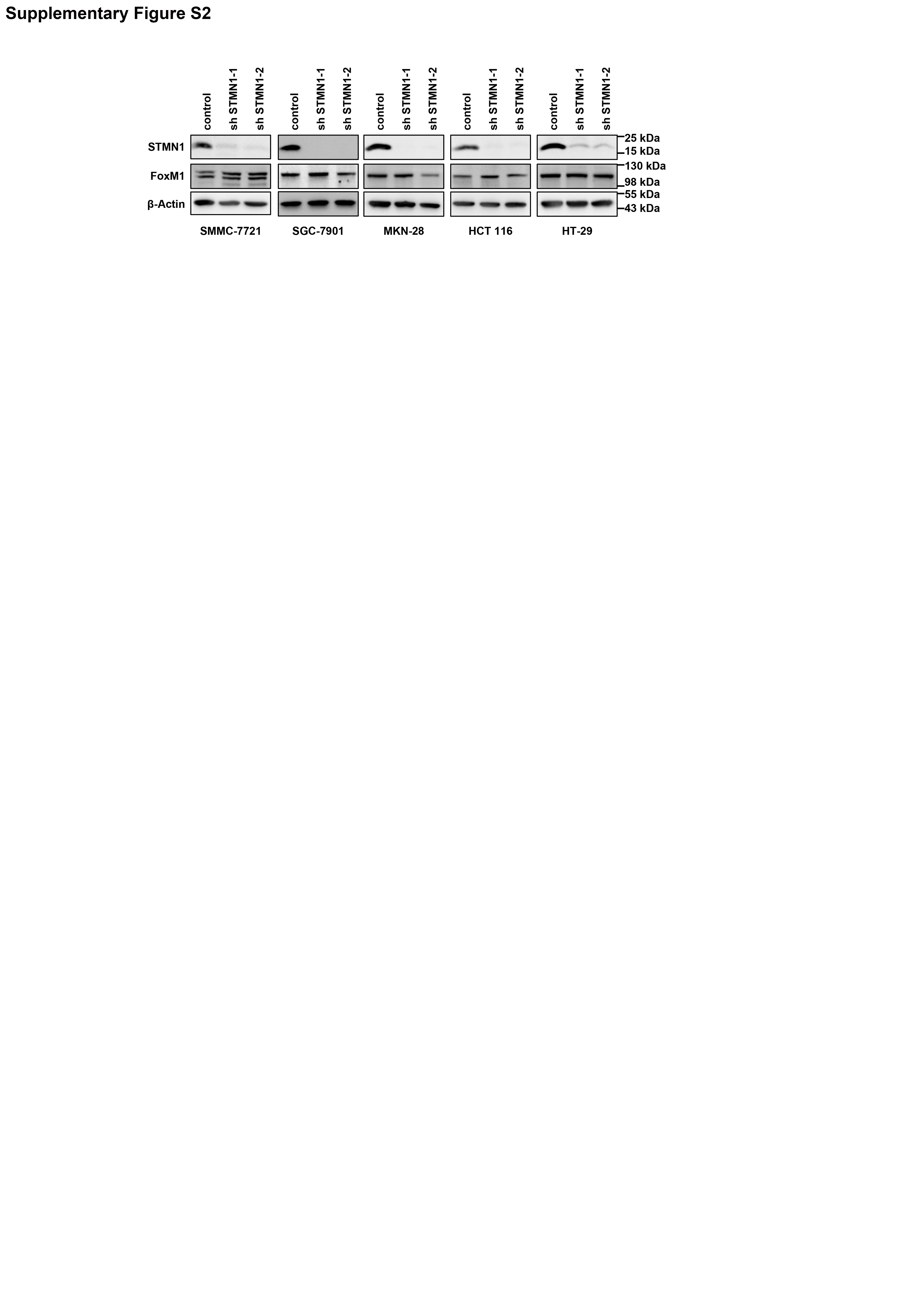

Supplement: Supplementary file 4 — Supplementary Figure S3 [file 41392_2020_396_MOESM4_ESM.tif]

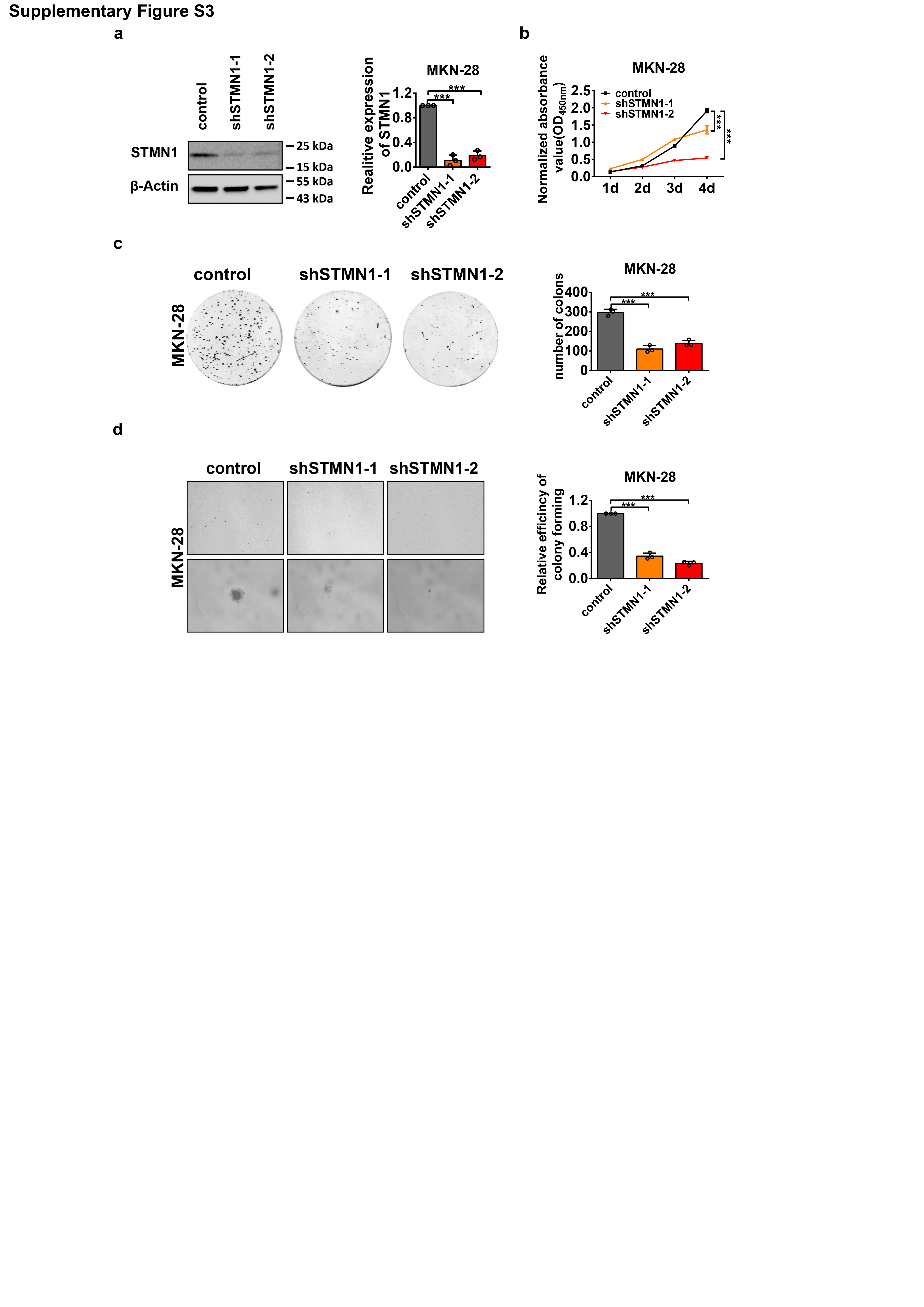

Supplement: Supplementary file 5 — Supplementary Figure S4 [file 41392_2020_396_MOESM5_ESM.tif]

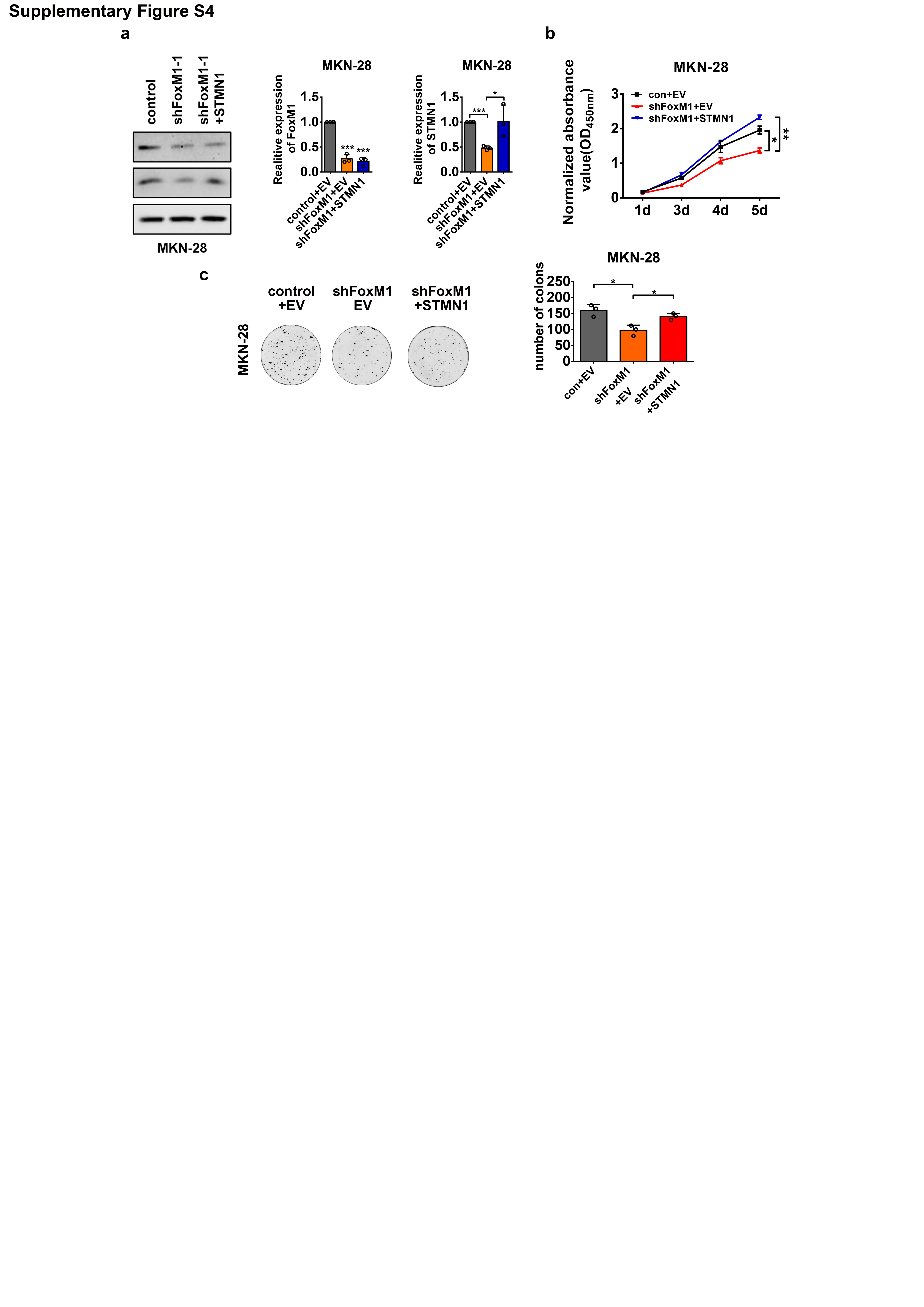

Supplement: Supplementary file 6 — Supplementary Figure S5 [file 41392_2020_396_MOESM6_ESM.tif]
