# Supplementary material for: Aberrantly high activation of a FoxM1–STMN1 axis contributes to progression and tumorigenesis in FoxM1-driven cancers
Source: Signal Transduct Target Ther. 2021 Feb 1;6:42. doi: 10.1038/s41392-020-00396-0 (PMC7851151; doi:10.1038/s41392-020-00396-0)
Supplement: Supplementary file 2 — Supplementary Figure S1 [file 41392_2020_396_MOESM2_ESM.docx]

Supplementary Materials for

Aberrantly High Activation of A FoxM1-STMN1 Axis Contributes to Progression and Tumorigenesis in FoxM1-Driven Cancers

Jun Liu ^1,2 #^, Jipeng Li ^2, 3 # *^, Ke Wang ^2 #^, Haiming Liu ^4 #^, Jianyong Sun ^5 #^, Xinhui Zhao ^6^, Yanping Yu ^7^, Yihuan Qiao ^8^, Ye Wu ^1^, Xiaofang Zhang ^1^, Rui Zhang ^1,9 *^, Angang Yang ^9 *^

Correspondence to: agyang@fmmu.edu.cn; ruizhang@fmmu.edu.cn; jipengli1974@aliyun.com

**This PDF file includes:**

Figures. S1 to S5

Tables S1 to S4

Figure. S1.

Tissue microarrays staining of FoxM1 and STMN1.


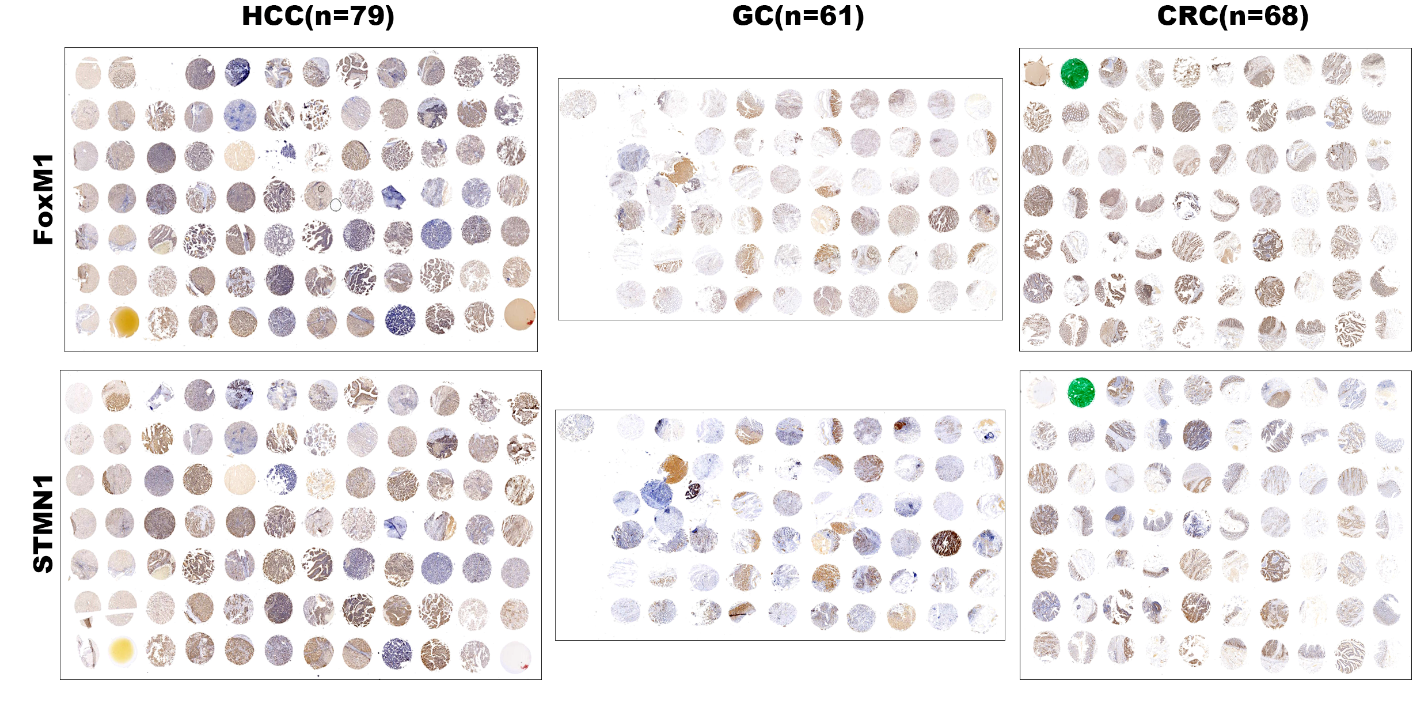


Tissue microarray staining of tumor samples of FoxM1 and STMN1 from 79 HCC, 61 GC and 68 CRC patients.

Figure. S2.

FoxM1 expression in STMN-knockdown cells.


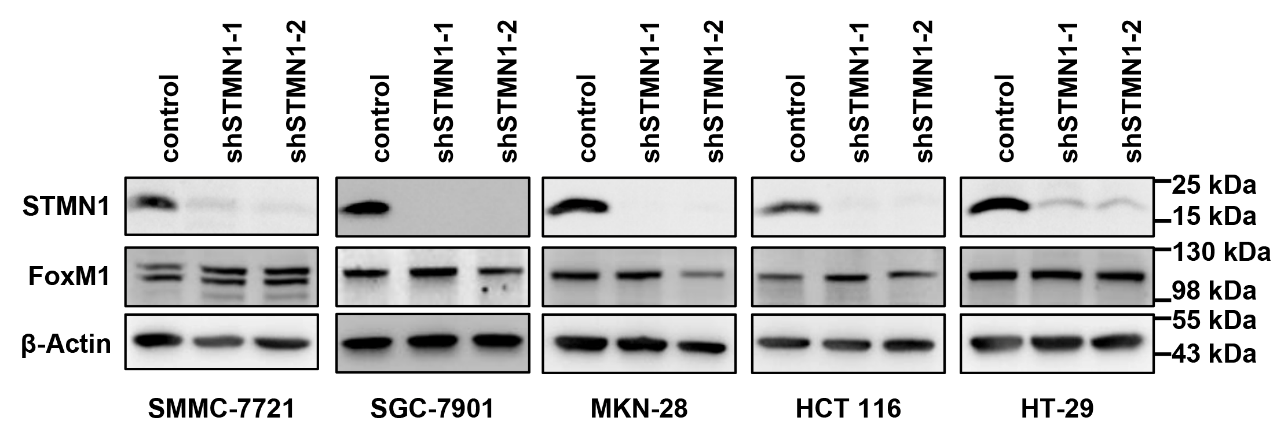


The hepatocellular carcinoma cell line SMMC-7721, gastric cancer cell lines SGC7-901 and MKN-28, colorectal cancer cell line HCT 116 and HT-29 were used to establish STMN1-knockdown cells using pLKO.1 gene silence system. The protein levels of STMN1 and FoxM1 were detected by Western blot.

Figure. S3.

STMN1 is essential for survival and proliferation in MKN-28 cell.


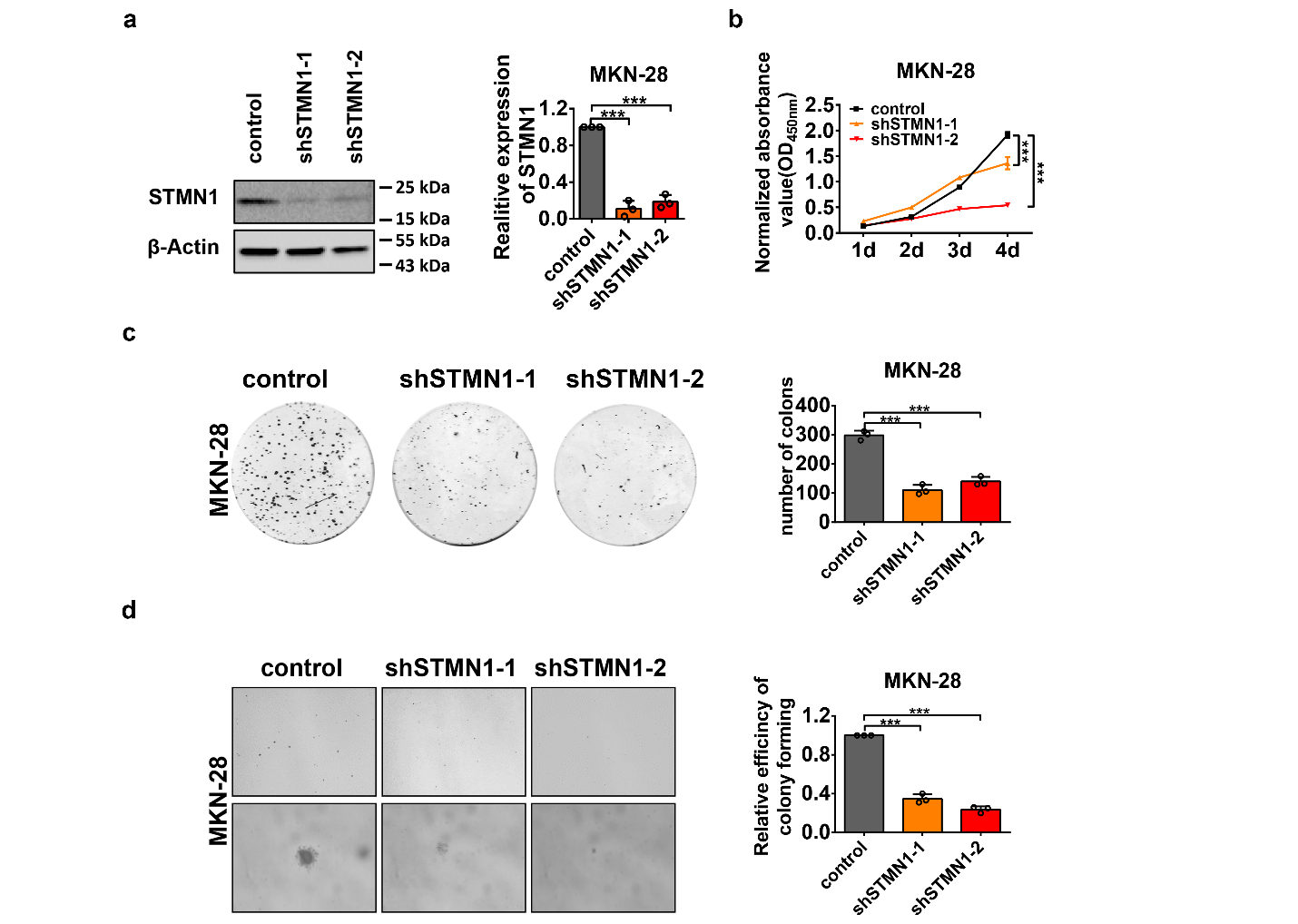


(a) The gastric cancer cell line MKN-28 was used to establish STMN1-knockdown cells using pLKO.1 gene silence system. The protein level of STMN1 was detected by Western blot, and the mRNA level was detected by RT-qPCR. The data were presented as the mean ± SD of three independent experiments. The significance was analyzed by student t-test. ***P < 0.001 (b) The cell viability of MKN-28 was detected by cell counting-8 kit (CCK-8). The data were presented as the mean ± SD of three independent experiments. The significance was analyzed by student t-test. ***P < 0.001 (c) The plate clone assay was performed and the number of clones was measured by ImageJ software. The data were presented as the mean ± SD of three independent experiments. The significance was analyzed by student t-test. ***P < 0.001 (d) The soft ager assay was performed and the tumorigenic spheres were photographed after 2 weeks since the cells were seeded in the 3D soft ager. The data were presented as the mean ± SD of three independent experiments. The significance was analyzed by student t-test. **P < 0.01, ***P < 0.001

Figure. S4.

FoxM1-mediated cancer cell proliferation requires STMN1 expression in MKN-28 cell.


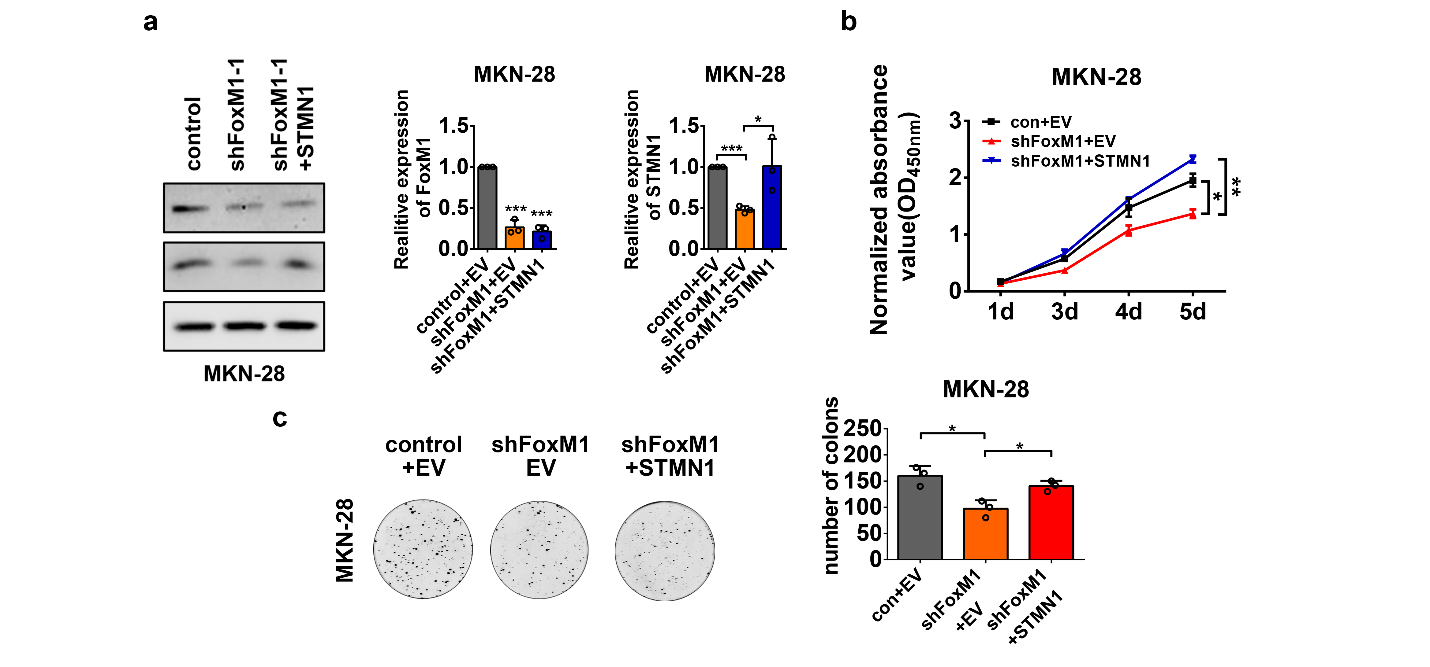


(a) The gastric cancer cell line MKN-28 was used to established FoxM1-silenced cell line by lentivirus mediated system. The protein levels of FoxM1 and STMN1 were detected by Western blot and the mRNA levels were detected by RT-qPCR. The data were presented as the mean ± SD of three independent experiments. The significance was analyzed by student t-test. *P < 0.05, **P < 0.01, ***P < 0.001 (b) The cell viability of cells was detected by cell counting-8 kit. The data were presented as the mean ± SD of three independent experiments. The significance was analyzed by student t-test. *P < 0.05, **P < 0.01, ***P < 0.001 (c) The plate clone assay was performed and the number of clones was measured by ImageJ software. The data were presented as the mean ± SD of three independent experiments. The significance was analyzed by student t-test. *P < 0.05, **P < 0.01

Figure. S5.

FoxM1-STMN1 signal axis is a risk of poor prognosis in GC cancer.


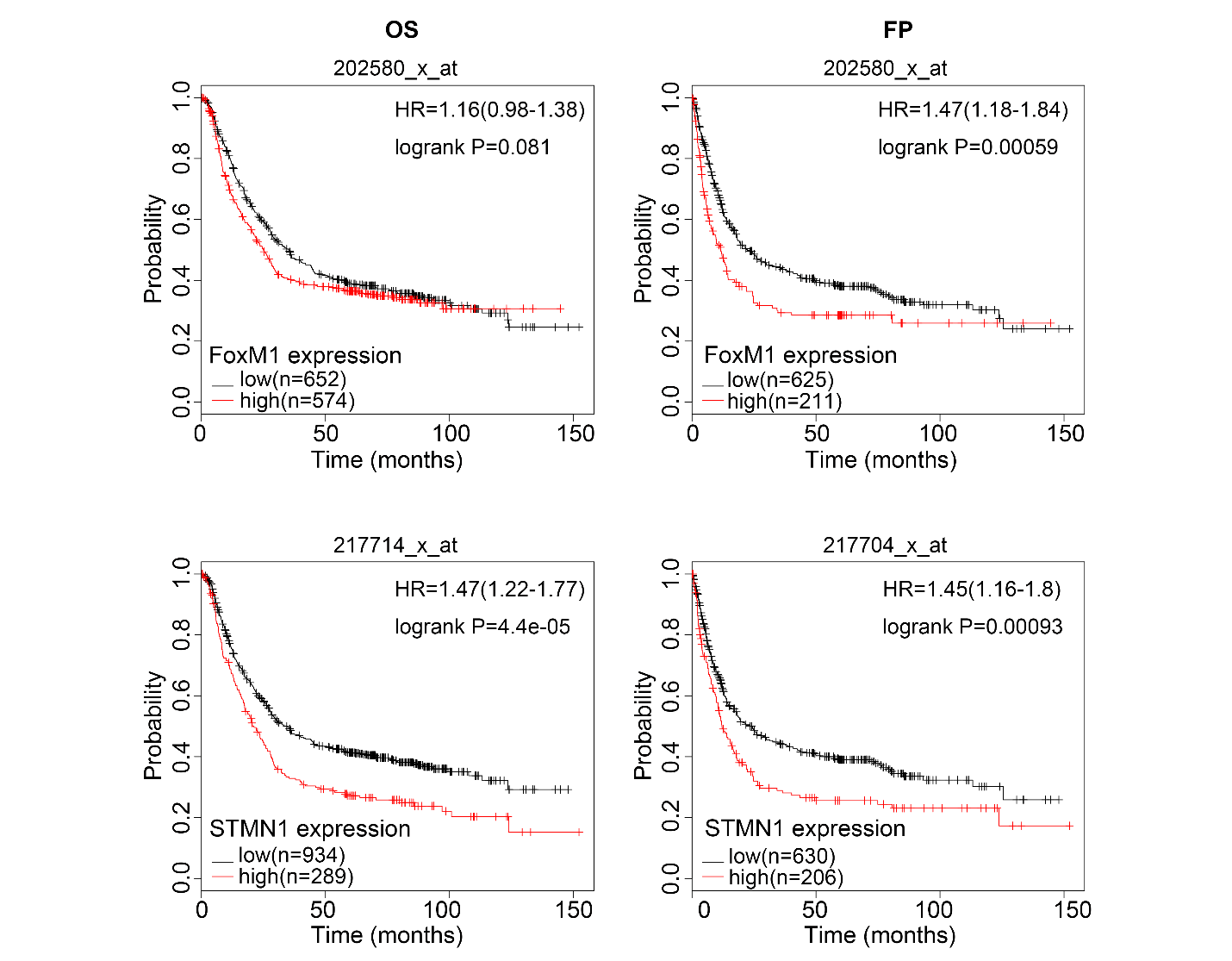


Overall survival and free-progression survival rate of gastric cancer patients were analyzed by KM-Plotter depending on expression of FoxM1 or STMN1.

Table S1.

Co-expression of FoxM1 and STMN1 using cBioPortal.

| cancer type | | Spearman | P value | Pearson | P value | samples |
| --- | --- | --- | --- | --- | --- | --- |
| THYM | Thymoma | 0.81 | 5.12E-29 | 0.84 | 1.68E-32 | 119 |
| ACC | Adrenocortical Carcinoma | 0.75 | 3.65E-15 | 0.79 | 3.02E-17 | 75 |
| BRCA | Breast Invasive Carcinoma | 0.73 | 9.52E-160 | 0.73 | 2.24E-158 | 960 |
| LIHC | Liver Hepatocellular Carcinoma | 0.73 | 1.67E-64 | 0.73 | 3.24E-63 | 373 |
| MESO | Mesothelioma | 0.64 | 3.49E-11 | 0.63 | 4.57E-11 | 87 |
| PAAD | Prostate Adenocarcinoma | 0.63 | 1.82E-55 | 0.67 | 4.53E-67 | 498 |
| KICH | Kidney Chromophobe | 0.62 | 2.98E-08 | 0.72 | 7.63E-12 | 66 |
| LUAD | Lung Adenocarcinoma | 0.57 | 7.43E-46 | 0.56 | 1.33E-42 | 510 |
| SARC | Sarcoma | 0.55 | 5.35E-22 | 0.59 | 4.31E-26 | 263 |
| STAD | Stomach Adenocarcinoma | 0.53 | 2.25E-31 | 0.56 | 3.09E-36 | 415 |
| GBM | Glioblastoma Multiforme | 0.5 | 1.31E-33 | 0.5 | 6.81E-35 | 521 |
| OV | Ovarian Serous Cystadenocarcinoma | 0.48 | 1.48E-32 | 0.47 | 8.64E-31 | 530 |
| CHOL | Cholangiocarcinoma | 0.47 | 3.49E-03 | 0.6 | 1.10E-04 | 36 |
| BLAC | Bladder Urothelial Carcinoma | 0.46 | 2.39E-22 | 0.51 | 3.27E-28 | 408 |
| ESCA | Esophageal Carcinoma | 0.44 | 3.05E-10 | 0.45 | 2.62E-10 | 183 |
| KIRC | Kidney Renal Clear Cell Carcinoma | 0.43 | 8.54E-26 | 0.49 | 4.83E-33 | 534 |
| TGCT | Testicular Germ Cell Tumors | 0.41 | 2.74E-07 | 0.44 | 3.90E-08 | 144 |
| THCA | Thyroid Carcinoma | 0.4 | 2.86E-21 | 0.46 | 3.19E-28 | 509 |
| LAML | Acute Myeloid Leukemia | 0.39 | 8.61E-08 | 0.51 | 8.99E-13 | 173 |
| UCEC | Uterine Corpus Endometrial Carcinoma | 0.36 | 2.10E-08 | 0.35 | 2.93E-08 | 232 |
| HNSC | Head and Neck Squamous Cell Carcinoma | 0.31 | 2.71E-13 | 0.31 | 9.14E-13 | 522 |
| PRAD | Pancreatic Adenocarcinoma | 0.3 | 5.27E-05 | 0.2 | 6.75E-03 | 179 |
| CRC | Colorectal Adenocarcinoma | 0.29 | 6.01E-09 | 0.3 | 1.66E-09 | 382 |
| SKCM | Skin Cutaneous Melanoma | 0.25 | 4.60E-08 | 0.25 | 6.74E-08 | 472 |
| UCS | Uterine Carcinosarcoma | 0.21 | 1.23E-01 | 0.19 | 1.53E-01 | 56 |
| LUSC | Lung Squamous Cell Carcinoma | 0.15 | 1.18E-03 | 0.15 | 9.78E-04 | 484 |
| CESC | Cervical Squamous Cell Carcinoma | 0.1 | 9.19E-02 | 0.09 | 1.11E-01 | 306 |
| DLBC | Diffuse Large B-Cell Lymphoma | -0.01 | 9.57E-01 | 0.06 | 7.08E-01 | 48 |
| LGG | Brain Lower Grade Glioma | -0.02 | 0.71 | 0.03 | 0.538 | 530 |
| PCPG | Pheochromocytoma and Paraganglioma | -0.02 | 7.96E-01 | 0.02 | 7.92E-01 | 178 |
| KIRP | Kidney Renal Papillary Cell Carcinoma | -0.07 | 2.66E-01 | -0.09 | 1.19E-01 | 274 |

Table S2.

Primers used for shRNA.

| Target | Sequences |
| --- | --- |
| *shFoxM1*-1 | CCGGGCCAATCGTTCTCTGACAGAACTCGAGTTCTGTCAGAGAACGATTGGCTTTTTG |
| *shFoxM1*-2 | CCGGGCCCAACAGGAGTCTAATCAACTCGAGTTGATTAGACTCCTGTTGGGCTTTTTG |
| *shSTMN1*-1 | CCGGGCTAATGTAGGACTGTATAGGCTCGAGCCTATACAGTCCTACATTAGCTTTTTG |
| *shSTMN1*-2 | CCGGGCACGAGAAAGAAGTGCTTCACTCGAGTGAAGCACTTCTTTCTCGTGCTTTTTG |

Table S3.

Primers used for qPCR.

| Gene symbol | Forward primer | Reverse primer |
| --- | --- | --- |
| *FoxM1* | ATACGTGGATTGAGGACCACT | TCCAATGTCAAGTAGCGGTTG |
| *STMN1* | TCAGCCCTCGGTCAAAAGAAT | TTCTCGTGCTCTCGTTTCTCA |
| *ACTIN* | CGGCACCACCATGTACCCTG | ACACGGAGTACTTGCGCTCA |

Table S4.

Primers used for ChIP-qPCR.

| Gene Name | Forward primer | Reverse primer |
| --- | --- | --- |
| *STMN1* | AACTCTTTAACATTTCACCGA | TTGGGCGTTTTCTTATCCAC |
| *Neagtive control* | AGTGGGTAAGGACCTCTTG | TGAACAGAATCTCAGACATCA |
